# Evolutionary diversity of sphingolipid metabolism proteins in fungi

**DOI:** 10.3897/imafungus.17.177891

**Published:** 2026-05-04

**Authors:** Magdalena Płecha, Blanka Sokołowska, Drishtee Barua, Julia Bartczak, Maria Sobczyk, Dagmara Koperska, Anna Kułakowska, Adam Komosa, Anna Muszewska

**Affiliations:** 1 Institute of Biochemistry and Biophysics, Polish Academy of Sciences, Pawinskiego 5A, 02-106 Warsaw, Poland Institute of Biochemistry and Biophysics, Polish Academy of Sciences Warsaw Poland; 2 Institute of Microbiology, ETH Zürich, Vladimir-Prelog-Weg 4, 8049 Zürich, Switzerland Faculty of Biology, University of Warsaw Warsaw Poland; 3 Doctoral School of Molecular Biology and Biological Chemistry at IBB PAS, Pawinskiego 5a, 02-106 Warsaw, Poland Institute of Microbiology, ETH Zürich Zürich Switzerland; 4 Faculty of Biology, University of Warsaw, Department of Molecular Biology, Laboratory of Microfluidics and Single Cell Biology, Miecznikowa 1, 02-096 Warsaw, Poland Warsaw University of Life Science - SGGW Warsaw Poland; 5 Warsaw University of Life Science - SGGW, Nowoursynoiwska 166, 02-787, Warsaw, Poland Doctoral School of Molecular Biology and Biological Chemistry at IBB PAS Warsaw Poland

**Keywords:** Ceramides, fungal adaptation, fungal evolution, lipid biosynthesis, phylogenetics, sphingolipids

## Abstract

Fungi are adapted to survive across environments with fluctuating oxygen availability. Transition to terrestrial and parasitic lifestyles required remodeling of the cell membrane, including sphingolipid composition. Ceramides occupy a central position in sphingolipid metabolism, a topic of growing biotechnological interest due to the high lipid-producing capacity of fungi. However, current knowledge of fungal metabolism is largely derived from *Dikarya*. Here, we characterize the repertoire of proteins involved in sphingolipid metabolism across the fungal tree of life and trace the evolutionary history of selected proteins. We identify 59 protein subfamilies, over half of which are widely conserved, while the remaining exhibit lineage-specific duplications and losses, suggesting roles in adaptive processes. Protein diversity is higher in lineages associated with aerobic conditions compared to anaerobic, often parasitic or endosymbiotic groups. Flagellated and non-flagellated fungi also differ, particularly in ceramide synthesis and sphingomyelin-processing enzymes. The divergence times of key sphingolipid-metabolizing enzymes, such as acid sphingomyelin phosphodiesterase and UDP-glucuronosyltransferase, correspond to the period of fungal terrestrialization in non-flagellated fungi, except in *Glomeromycota* and *Dikarya*, where the divergence of acid sphingomyelin phosphodiesterase coincides with the evolutionary radiation of flowering plants. These findings show the diversification of sphingolipid metabolism, suggesting its role in adaptation and membrane specialization during fungal evolution.

## Introduction

Fungal adaptation to diverse habitats involves coping with physical constraints, such as oxygen availability and fatty acid supply. The transition to terrestrial or parasitic lifestyles therefore required the evolution of mechanisms conferring resistance to adverse environmental conditions. Among these adaptations, sphingolipid biosynthesis represents a key trait that diversified during fungal terrestrialization (Wu et al. 2022).

Fungi inhabiting soil, rock surfaces, or living hosts are frequently exposed to water imbalance and osmotic stress (Dupont et al. 2011). In contrast, aquatic fungi must cope with low water potential, salinity, and pH fluctuations—challenges partly mitigated by hydrophobic cell walls (Hernández-Saavedra, Ochoa, and Vazquez-Dulhalt 1995; Arias-Real et al. 2023). The fungal cell membrane, supported by the cell wall, forms the primary barrier to environmental stress. In many terrestrial taxa, incorporation of ergosterol into the plasma membrane enhances mechanical resilience against osmotic fluctuations (Dupont et al. 2011). Parasites, endosymbionts, and certain pathogens, however, rely on diverse strategies to withstand anaerobic or microaerophilic conditions. Whereas oxygen sensing in animals is largely mediated by the hypoxia-inducible factor (HIF) pathway, fungi lack direct homologs and have instead evolved distinct regulatory mechanisms, particularly involving lipid turnover, including sterol and glycerophospholipid metabolism. In contrast to sterols, sphingolipid metabolism remains less understood, highlighting the need to expand knowledge of fungal adaptation to low-oxygen environments (Burr and Espenshade 2018; Hess et al. 2020).

Sphingolipids (SL) are a class of lipids comprising ceramides (Cer), sphingosine (SPH), sphingosine-1-phosphate (S1P), and ceramide-1-phosphate (C1P) (Mao and Obeid 2008; Hannun and Obeid 2017). They constitute a major component of eukaryotic plasma membranes and play essential roles in growth, survival, differentiation, and senescence, as well as in inflammation, immune responses, and stress adaptation (Santos et al. 2020). Although present in relatively low abundance and lacking a glycerol backbone, sphingolipids are pivotal for cellular homeostasis and signaling (Hannun and Obeid 2017).

Ceramides are a subclass of sphingolipids, defined by a sphingosine or related long-chain base linked to a fatty acid via an amide bond (Alonso and Goñi 2018). Compared to other membrane lipids, ceramides are highly hydrophobic (Alonso and Goñi 2018) and occupy a central position in sphingolipid metabolism. Their synthesis and regulation depend on subcellular localization; for example, ceramide synthase (CerS) resides in the endoplasmic reticulum, whereas inositol phosphorylceramide synthase 1 is localized to the Golgi apparatus (McEvoy et al. 2020). The de novo ‘entry pathway’ begins with serine palmitoyltransferase (SPT), which condenses palmitate and serine to form 3-keto-dihydrosphingosine, subsequently reduced to dihydrosphingosine (sphinganine, DHS). Next, CerS acylates DHS, which is followed by desaturation to form ceramide. Human CerS isoforms, numbered CERS1–6, display distinct acyl-chain selectivity (Wang et al. 2024). Ceramide can be further converted into various bioactive lipid species, including ceramide-1-phosphate via ceramide kinase (CK), sphingomyelin (SM) through sphingomyelin synthase (SMS), sphingosine by ceramidase (CDase), and glucosylceramide (GluCer) via glucosylceramide synthase (GCS). These metabolites are also interconnected with glycosphingolipids (GSL), forming a complex network of sphingolipid derivatives (Hannun and Obeid 2011).

Glycosphingolipids are glycolipids composed of a ceramide backbone, in which a sphingoid base is linked to a fatty acid via an amide bond at C-2. In fungi, phytosphingosine (PHS) is the predominant sphingoid base (Varki et al. n.d.). GSLs are essential constituents of the plasma membrane, enriched in the outer leaflet, where they modulate signal transduction, cell adhesion, growth, cell cycle progression, and apoptosis (Guimarães et al. 2014). They are broadly classified into neutral GSL, such as glucosylceramide and galactosylcera (Varki et al. n.d.) mide (GalCer), and acidic GSL, including glycosyl inositol phosphoceramide (GIPC), mannosyl inositol phosphorylceramide (MIPC), and mannose inositol phosphoryl-2-ceramide (M(IP)_2_C) (Buré et al. 2014).

Sphingolipids, including glycosphingolipids and ceramides, have been primarily studied in model *Ascomycota* and *Basidiomycota*, such as *Yarrowia lipolytica* (Jiang et al. 2021; Han et al. 2021). Neutral GSL, including GluCer and GalCer, is common in filamentous Asco- and Basidiomycota. In contrast, yeasts predominantly produce glycosyl inositol phosphoceramide, which is lacking in some non-*Dikarya* fungi (Mandala and Harris 2000; Aoki et al. 2004; Guimarães et al. 2014; Fernandes et al. 2018; McEvoy et al. 2020). In *Dikarya*, ceramides synthesized in the ER are transported to the Golgi, serving as precursors for GluCer and inositol phosphorylceramide (IPC) (Mota Fernandes and Del Poeta 2020). Unlike other fungi, *Saccharomycotina* lack glucosylceramide synthase and instead rely on IPC synthase (IPCS), IPC mannosyltransferase (IPCT), and inositolphosphotransferase (IPT) for the biosynthesis of inositol-containing complex sphingolipids, including MIPC and M(IP)_2_C (Lu et al. 2020). Additionally, certain non-*Dikarya* fungi, such as *Mortierella alpina*, produce diverse ceramides and ceramide-1-phosphates, which account for over 80% of their sphingolipid pool (Wang et al. 2024).

Despite the recognized biotechnological relevance of ceramides and related sphingolipids, a comprehensive characterization of the fungal enzymes involved in their biosynthesis is still lacking. Existing studies and reviews have predominantly focused on *Dikarya* (Usmani et al. 2023), whereas research on non-model fungi remains fragmentary and often inconsistent with experimental evidence. For several compounds, biosynthetic pathways have not yet been elucidated, even though the corresponding lipids have been isolated from fungal mycelia.

To address this knowledge gap, we conducted an extensive literature survey and sequence-based screening across a broad set of fungal proteomes to identify candidate genes encoding ceramide- and sphingolipid-associated proteins. The resulting dataset was analyzed from taxonomic, ecological, and molecular perspectives using phylogenomic inference and molecular dating. This approach enabled us to comprehensively characterize the evolutionary trajectories of sphingolipid-related traits across the fungal tree of life, with particular emphasis on contrasting flagellated and non-flagellated lineages and linking their metabolic repertoires to lifestyle diversity and oxygen availability.

## Methods

### Homologous sequence search

Protein sets of all then-available early diverging fungi (*n* = 148) and representatives of 35 classes of *Dikarya*, referred to as the ‘target proteomes’ (Suppl. material 1), were downloaded from NCBI in May 2022 (NCBI Resource Coordinators 2018). Genome completeness and quality were previously assessed by (Steczkiewicz et al. 2025) using BUSCO v. 5.7.1 against the fungi_odb10.

The starting point of this study was to create an initial list of enzymes involved in ceramide metabolism across *Opisthokonta*. We searched the UNIPROT (UniProt Consortium 2021) and REACTOME version 83 (Gillespie et al. 2022) databases using the following keywords: sphingolipid, sphingomyelin, ceramide, galactosylceramide, glucosylceramide, glycosphingolipids, phospholipid, and steroid. The retrieved proteins were grouped into families based on their domain composition, with all proteins sharing the same Pfam domain assigned to the same family. In the case of multidomain proteins, we used the catalytic domain to define family membership. Based on previous studies, we selected protein families associated with sphingolipid and ceramide metabolism. For each protein, we selected reference sequences that served as queries for searches against the target proteomes (Suppl. material 1). Fungal homologs sharing protein domains were analyzed together. Each protein set was clustered in CLANS using *p*-value thresholds of 1 × 10^−5^, 1 × 10^−15^, and 1 × 10^−20^, with the expulsive force exponent set to two and other parameters left at default values (Frickey and Lupas 2004). To eliminate false positives, we analyzed the resulting clusters in terms of putative protein function based on domain architectures predicted by PFAMSCAN.PL (run with default parameters against the PFAM databases v. 33 and v. 36) (Mistry et al. 2021) and INTERPROSCAN 5.60–92.0 (Jones et al. 2014). Each group of homologs was extracted from the CLANS clustering and used to count their occurrences in the target proteomes. Finally, we performed phylogenetic analyses for homolog groups showing the largest differences in counts, which may indicate evolutionary events such as gene duplications or losses.

### Phylogenetic tree and divergence time estimation

We used three types of protein sequences to calculate phylogenetic trees: 1) fungal sequences (derived from 183 target proteomes), 2) basal opisthokont sequences (derived from BLASTP searches of reference sequences against the *Opisthokonta* proteomes), and 3) metazoan and fungal (e.g., *Saccharomyces cerevisiae*) reference sequences retrieved from UNIPROT. We aligned collected amino acid sequences using the local iterative alignment method in MAFFT v. 3.7 (--localpair, --maxiterate 1000, --reorder) (Katoh et al. 2002) and manually trimmed in JALVIEW to the conserved domain region if necessary (Waterhouse et al. 2009) or automated trimming using TRIMAL (--gappyout) (Capella-Gutiérrez et al. 2009). We constructed trees based on the maximum likelihood (ML) method using IQ-TREE 2.0.3 (Minh et al. 2020) with prediction of the best model for phylogenetic analysis (*m* = MFP). Dating and calibration of the trees were performed with the least squares dating (LSD2) (To et al. 2016) method (applied in IQ-TREE 2.0.3). In the datefile (--date), we implied the following calibration time priors for the tips: basal opisthokonts – 1083 Mya, animals – 792 Mya, *Rozellomycota* – 792 Mya, older lineages of non-*Dikarya* – 646 Mya, *Zoopagomycota* – 671 Mya, *Mortierello-*, *Glomero-*, and *Mucoromycota* (crown) – 582 Mya, *Dikarya* – 411 Mya (Liu et al. 2024). As an outgroup, we included homologous sequences from basal opisthokonts and animals (e.g., human, mouse, or fruit fly) (Suppl. material 1). We calculated two categories of ML trees. The first set of trees was built to confirm the distribution of analyzed protein subfamilies and included all fungal protein sequences found for selected traits. The second set of trees served to calculate the timescale of evolutionary events and included only selected fungi representing all main lineages in fungi (*Rozello-*, *Neocallimastigo-*, *Chytridio-*, *Blastocaldio-*, *Zoopago-*, *Mucoro-*, *Mortierello-*, *Glomeromycota*, and *Dikarya*). We computed a phylogenomic tree for species representing fungal phyla with ORTHOFINDER 2.5.5, using MMSEQS2 as the search engine (Emms and Kelly 2019). We visualized and annotated all ML and dated trees in ITOL v. 7 (Waterhouse et al. 2009; Letunic and Bork 2021).

### Transcriptomic data analysis

In order to verify if the genes encoding the analyzed proteins are expressed in diverse environmental conditions, we searched for publicly available transcriptomic datasets of non-*Dikarya* species. The dataset consisted of transcriptomes of *Mucoro-* and *Mortierellomycota* grown in axenic cultures, along with transcriptomes obtained from diverse environments in *Mucoro-* and *Glomeromycota*. The RNA-Seq data were downloaded in the form of fastq files from the ENA server (Leinonen et al. 2011). The fastq files were quality checked using FASTQC (v. 0.11.8) (Andrews and Others 2010), followed by adapter trimming using FASTP (v. 0.19.6) (Chen et al. 2018) with default parameters. The trimmed reads were aligned with the reference genome of the respective organism (downloaded from NCBI Datasets) using HISAT2 (v. 2.1.0) (Kim et al. 2015; Chen et al. 2018). The alignment produced reads in the form of large SAM files that were compressed into binary file format (BAM) files using SAMTOOLS (v. 1.10) (H. Li et al. 2009). The aligned read (BAM) files were subjected to read counting against the GTF files of the respective species using FEATURECOUNTS (v. 1.6.3) (Liao et al. 2014). The STRINGTIE (v. 2.1.3b) (Pertea et al. 2015) tool was used to determine the transcript per million (TPM) reads from the BAM files of whole transcriptome datasets.

For analysis of transcriptomes obtained from diverse environments, differential expression analysis was carried out using the DESEQ2 R package (Love et al. 2014). The standard filtering parameters [Padj ≤ 0.05; (log2fold change: downregulation < 0 > upregulation)] were used to determine the gene expression profiles. In the case of whole transcriptomes, TPM > 1 was used to filter out the significant genes encoding proteins.

### List of abbreviations

**A3GALT2** Alpha-1,3-galactosyltransferase 2

**ACER1/2/3** Alkaline ceramidase 1–3

**ASAH1, Asah1** Acid ceramidase

**ASAH2, Asah2** Neutral ceramidase

**ASAH3, Asah3** Alkaline ceramidase

**ASMase** Acid sphingomyelin phosphodiesterase

**ATG26** Sterol 3-beta-glucosyltransferase

**B3GALT6** Beta-1,3-galactosyltransferase 6-like

**B4GALT2** Beta-N-acetylglucosaminyl glycolipid beta-1,4-galactosyltransferase

**C1P** Ceramide-1-phosphate

**CDase** Ceramidase

**Cer** Ceramide

**CerS** Ceramide synthase

**CERS1–6/LAC1/**

**LAG1, CerS** Ceramide synthase 1–6/Ceramide synthase LAC1/Ceramide synthase LAG1

**CK** Ceramide kinase

**DAG** Diacylglycerol

**DGAT1/SOAT1** Diacylglycerol O-acyltransferase 1

**DGAT2** Diacylglycerol O-acyltransferase 2

**DGK1/A, DAGK** Diacylglycerol kinase

**DEGS1** Sphingolipid delta4-desaturase (DES1)/acyl-lipid delta8(3)-desaturase

**DEGS2** Sphingolipid delta4-desaturase/C4-monooxygenase (DES2)

**DES1** Sphingolipid delta4-desaturase/acyl-lipid delta8(3)-desaturase

**DES2** Sphingolipid delta4-desaturase/C4-monooxygenase

**DHS** Dihydrosphingosine (sphinganine)

**DHS-1P** Dihydrosphingosine-1-phosphate

**EDF1-like** Very-long-chain fatty acid elongase subfamily 2

**EDF2-like** Very-long-chain fatty acid elongase subfamily 3

**ELOVL1/3** Very-long-chain fatty acid elongase family 1–3

**ERG3** C-5 sterol desaturase

**ERG25/MSMO1** Sterol C-4 methylsterol oxidase

**ERG27** Steroid reductase

**FADS1/DES1** Acyl-CoA delta-8(3)-desaturase

**FADS2** Acyl-CoA delta-6-desaturase

**FADS3** Fatty acid desaturase 3

**GBA1** Lysosomal acid glucosylceramidase

**GCS** Glucosylceramide synthase

**GIPC** Glycosyl inositol phosphoceramide

**GluCer** Glucosylceramide

**GalCer** Galactosylceramide

**GSL** Glycosphingolipid

**GT** Glycosyltransferase

**GT1** UDP-glycosyltransferases

**HIF** Hypoxia-inducible factor

**Ipc1** Inositol phosphoryl ceramide synthase 1

**IPC** Inositol phosphorylceramide

**IPCS** Inositol phosphorylceramide synthase

**IPCT**IPC mannosyltransferase

**IPT** Inositolphosphotransferase

**IPT1** Inositolphosphotransferase 1

**KDSR** 3-dehydrosphinganine reductase

**KES1** Oxysterol-binding protein homolog 4

**LCB3** Dihydrosphingosine-1-phosphate phosphatase

**LCB4/5** Long-chain base kinase

**LET-767** Short-chain dehydrogenase/3-ketoacyl-CoA reductase

**LIP1** Ceramide synthase regulatory subunit LIP1

**LPIN1** Phosphatidate phosphatase

**M(IP)_2_C** Mannose (inositol phosphoryl)_2_-ceramide

**MIPC** Mannosyl inositol phosphorylceramide

**MTS/MT1/MT2** Sphingolipid C9-methyltransferases

**NEU2** Sialidase

**NSMase** Neutral sphingomyelin phosphodiesterase

**OCH1** Alpha-mannosyltransferase

**OSH2/3** Oxysterol-binding protein homologs 2–3

**PA** Phosphatidic acid

**PHS** Phytosphingosine

**PHS-1P** Phytosphingosine-1-phosphate

**PLA2G4A** Lysophospholipase/Phospholipase A2

**PLPP1** Phospholipid phosphatase

**S1P** Sphingosine-1-phosphate

**SCD5** Acyl-CoA desaturase/delta9-fatty acid desaturase

**SCS7** Ceramide very-long-chain fatty acid hydroxylase

**SGPL1** Sphingosine-1-phosphate lyase

**SL** Sphingolipid

**SM** Sphingomyelin

**SMS** Sphingomyelin synthase

**SMS1/2** Sphingomyelin synthase 1–2

**SMPD1** Acid sphingomyelin phosphodiesterase

**SMPD2/3** Neutral sphingomyelin phosphodiesterase 2–3

**SMPD4** Sphingomyelin phosphodiesterase 4

**SPH** Sphingosine

**SPHK** Sphingosine kinase

**SPHK1/2** Sphingosine kinase 1–2

**SPT** Serine palmitoyltransferase

**SPTC1** Serine palmitoyltransferase subunit 1

**SPT2/3** Serine palmitoyltransferase subunits 2–3

**SRD5A3** Polyprenol reductase

**SUR1** Mannosylphosphorylinositol ceramide synthase

**SUR2** Sphingolipid C4-hydroxylase

**SUR4** Delta-6 elongase

**TECR** Very-long-chain enoyl-CoA reductase

**UGCG** Ceramide glucosyltransferase

**UGGT1** UDP-glucose:glycoprotein glucosyltransferase 1

**UGT8/CGT** UDP-glucuronosyltransferase/N-acylsphingosine galactosyltransferase

**WSD1** Wax ester synthase

**YEGS** Sphingoid long-chain lipid kinase

## Results

### Selected proteins in SL metabolism

In the following paragraphs, we navigate over the sphingolipid pathways starting from the *de novo* synthesis of ceramides, through ‘recycling’ and cooperating pathways, concluding with modifications of SL and their derivatives. While describing traits, we focus on the diversity in protein family distribution between non-flagellated and flagellated lineages, *Dikarya* and non-*Dikarya*, and correlate these differences with fungal ecological traits, including oxygen requirements.

**Figure 1.**
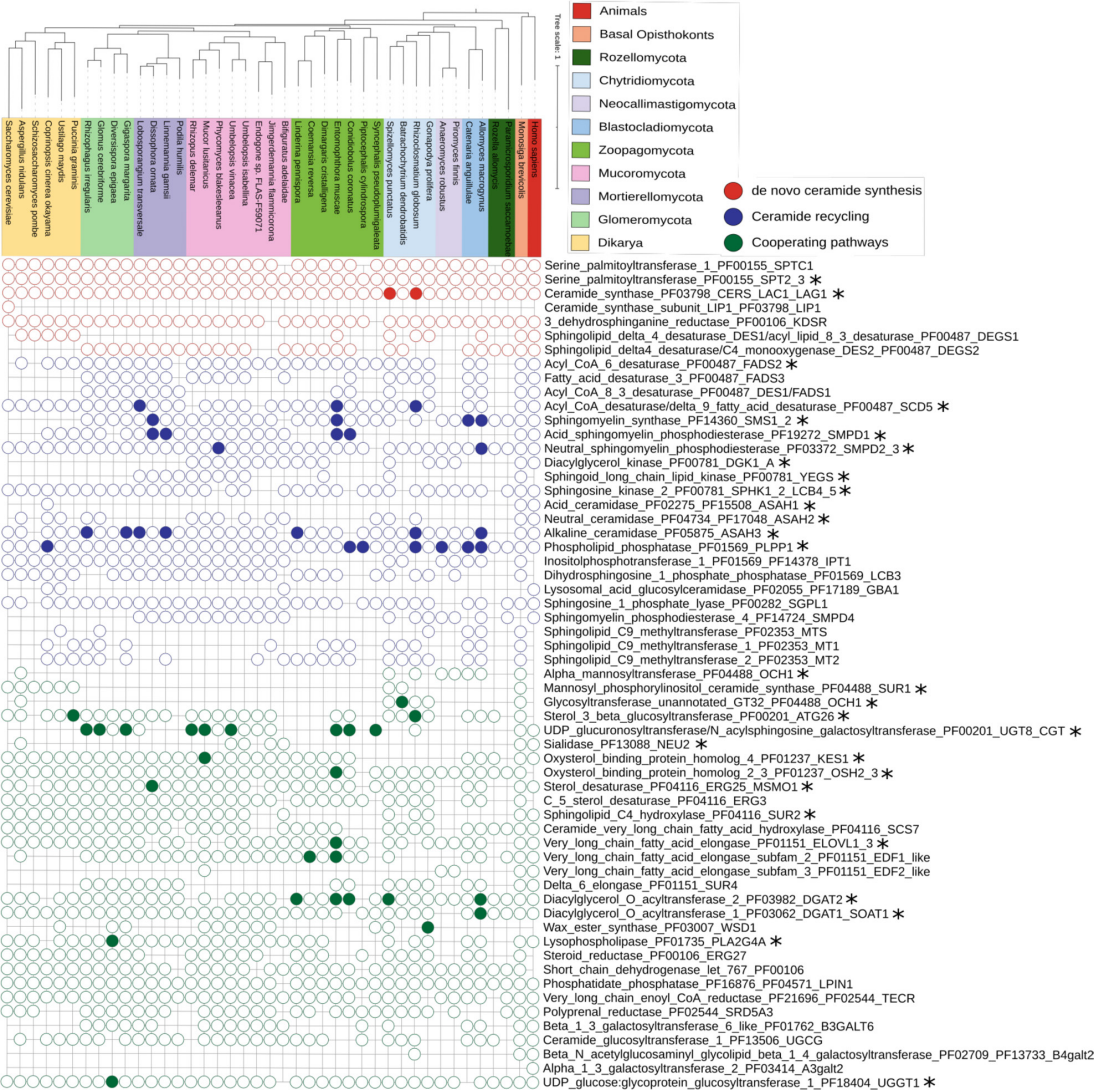
Gene duplications and losses shape the distribution of 59 sphingolipid metabolism proteins across fungi. The phylogenomic tree was computed with Orthofinder (Emms and Kelly 2019). Taxonomic distribution was drawn in ITOL (Waterhouse et al. 2009; Letunic and Bork 2021). Filled dots – five copies and more; empty dots – 1–4 copies; empty space – no homologs found. Protein names are structured as follows: the enzyme name is followed by the corresponding Pfam family identifier and the gene name(s). * – protein families potentially linked to adaptation to diverse environments.

We refer to *Chytridio-*, *Neocallimastigo-*, and *Blastocladiomycota* as ‘flagellated fungi’ and to *Mucoro-*, *Mortierello-*, *Glomero-*, *Zoopago-*, *Entomophthoro-*, *Kickxello-*, *Asco-*, and *Basidiomycota* as “non-flagellated fungi.” In our study, we consider Pfam families as the definition of a protein family. Pfam families are based on sequence and structure similarity, and members of a single family may differ in substrate specificity. In consequence, some of the protein families in our dataset needed to be categorized into subfamilies (for instance, the DGK protein family PF00781 has the following subfamilies: diacylglycerol kinase, sphingosine kinase, and sphingoid long-chain kinase/lipid YegS kinase). Most of the protein families with observable duplications and losses have more than one subfamily, which corresponds to different enzymatic functions or substrate specificity (Fig. 1).

### Distribution of protein subfamilies involved in ceramide metabolism varies across fungi

To assess the complexity of the metabolism of sphingolipids and related compounds across fungi, we searched a dataset of 183 fungal proteomes representing fungal diversity from *Rozellomycota* to *Dikarya* for the presence of 34 Pfam protein families, which we divided into 59 subfamilies. All subfamilies can be divided into three main pathway groups: *de novo* ceramide synthesis (seven subfamilies), ceramide recycling (22 subfamilies), and cooperating pathways (30 subfamilies). Studied proteins can be further assigned into six functional categories: fatty acid metabolism (15 subfamilies), sterol/lipid metabolism (seven subfamilies), glycoprotein metabolism (13 subfamilies), complex sphingolipid metabolism (seven subfamilies), ceramide biosynthesis (15 subfamilies), and lipid transfer (two subfamilies). Most of the analyzed protein families (33 out of 34) catalyze enzymatic reactions, whereas one is responsible for lipid transport (Suppl. material 1).

Analyzed subfamilies differ in taxonomic distribution, ranging from ubiquitous housekeeping enzymes involved in the central metabolism of lipids to lineage-specific ones. However, none of the phyla has all of the 59 analyzed subfamilies. Five out of seven *de novo* ceramide synthesis subfamilies and 19 out of 30 cooperating pathway components are conserved across all fungi, whereas about half (12/22) of the ceramide recycling proteins vary between phyla. For instance, phosphatidate phosphatase (converting phosphatidic acid to diacylglycerol) and ceramide synthase (responsible for acylation of sphingoid bases to ceramides) are found across all fungi. Similarly, alkaline ceramidase is ubiquitous in fungi, whereas acid and neutral ceramidases are rare. Among acetyltransferases, wax ester synthase is the least conserved, suggesting limited wax ester metabolism (Wenning et al. 2017), while diacylglycerol *O*-acyltransferase 1 (PF03062, DGAT1/SOAT1), essential for triacylglycerol synthesis, is broadly conserved (Suppl. material 1). There are other examples of protein families limited to a single lineage, for instance, beta-1,4-galactosyltransferase 2, transferring galactose onto N-glycans in *Rhizoclosmatium globosum*; alpha-1,3-galactosyltransferase 2, adding alpha-1,3-linked galactose to glycosphingolipids in *Neocallimastigomycota*; and ceramide synthase subunit LIP1, responsible for localization of LAG1 to the nuclear ER in *Saccharomycotina*. It is noteworthy that many of the subfamilies with limited taxonomic distribution are involved in glycoprotein modification, complex sphingolipid, and fatty acid metabolism. The most abundant glycoprotein-modifying enzymes in our set are sphingolipid glycosyltransferases, which, among others, modify compounds in the cell wall and are important for cell recognition and masking (Fig. 1, Suppl. material 1).

In total, across the 59 subfamilies, 29 show gene duplications in fungi. Some of these gene duplications may be a sign of adaptation to diverse environments, including terrestrialization and parasitism. For instance, the unusually large genome of *Entomophthora muscae* is enriched in genes encoding acyl-CoA desaturase/delta 9-fatty acid desaturases, sphingomyelin synthases, acid sphingomyelin phosphodiesterases, oxysterol-binding proteins, very long-chain fatty acid elongases, diacylglycerol *O*-acyltransferases 2, and UDP-glucuronosyltransferases, the latter having the highest copy number among all analyzed organisms. However, not all analyzed genes are equally copious in *E. muscae*, and most of the families are represented by fewer than 1–4 copies, suggesting a particular role of the duplicated ones (Fig. 1, Suppl. material 1).

The highest diversity of protein subfamilies occurs in *Glomeromycota* and *Mucoromycota* (44/59), followed by *Chytridio-* (42/59), *Blastocladio-* (41/59), and *Mortierellomycota* (40/59). About 50–60% are present in *Entomophthoro-*, *Basidio-*, *Zoopago-* (each 38/59), and *Ascomycota* (37/59). A lower diversity is seen in *Kickxellomycota* (31/59), *Neocallimastigomycota* (26/59), and *Rozellomycota* (24/59). *Olpidium bornovanus* encodes only 19/59 subfamilies, though this may reflect poor genome quality (BUSCO score 27%; it is the sole sequenced representative of the lineage). Microsporidia retain just 9/59, preserving only core pathways (e.g., *de novo* ceramide and fatty acid synthesis) while losing complex SL metabolism, which is likely an adaptation to anaerobic parasitism (Fig. 1, Suppl. material 1).

Most of the analyzed subfamilies are shared with animals, suggesting their ancestral *Opisthokonta* origin. There are, however, exceptions, such as *Saccharomycotina*LIP1; mannosyl-specific glycosyltransferases (OCH1 and SUR1) found only in *Dikarya* and flagellated fungi; and sterol 3-beta-glucosyltransferase (ATG26) found in most phyla, except *Kickxello-*, *Neocallimastigo-*, *Chytridio-*, and Microsporidia. Housekeeping oxysterol-binding protein homologs 2 and 3, involved in sterol trafficking and membrane balance, are also fungal-specific. The C-5 sterol desaturase (ERG3, PF04116) is similarly restricted to fungi and lacks direct homologs in animals. In contrast, other sterol biosynthetic genes show broader conservation: C-4 methylsterol oxidase (ERG25, PF04116; ortholog of human MSMO1) and 3-keto-steroid reductase (ERG27, PF00106; ortholog of human HSD17B7) are widely distributed across fungal and animal lineages. Altogether, these patterns underscore the evolutionary diversification and broad distribution of ERG genes (Suppl. materials 1, 2).

Fungi are not homogeneous, and one of the major differences in SL protein distribution is observed between *Dikarya* and non-*Dikarya*. *Asco-* and *Basidiomycota* appear to have lost some enzymes shared by non-*Dikarya* and animals, including sphingomyelin synthase, diacylglycerol kinase (ATP-dependent), sphingomyelin phosphodiesterase 4, beta-1,3-galactosyltransferase 6, and UDP-glucuronosyltransferase. Sphingomyelin synthase and sphingomyelin phosphodiesterase are key enzymes involved in sphingomyelin biosynthesis in the *Opisthokonta* lineage, a metabolic feature apparently lost in *Dikarya*. They also lack typical non-*Dikarya* proteins, for instance, sphingoid long-chain lipid kinase YEGS (Fig. 1, Suppl. material 1).

*De novo* ceramide synthesis appears to be conserved among opisthokonts, indicating its origin as an ancestral metabolic feature. However, compared to early diverging eukaryotes, animals, and non-motile fungi, flagellated fungi have undergone a reduction in the diversity of SL recycling and associated enzymes, such as acid sphingomyelin phosphodiesterase, acid ceramidase, lysosomal acid glucosylceramidase, sphingolipid C9-methyltransferase, beta-1,3-galactosyltransferase 6, UDP-glucuronosyltransferase, oxysterol-binding protein homolog 4, C5-sterol desaturase, and steroid reductase, suggesting these proteins may not be essential in aquatic habitats (Fig. 1, Table 1, Suppl. material 1).

**Table 1.** Sphingolipid metabolism-related genes with most contrasting abundance between flagellated and non-flagellated fungal lineages. +/- present in some representatives, + present, and ++ duplicated.

Gene	Flagellated fungi	Non-flagellated fungi	Pathway
LAC1/LAG1	+/-	++	Ceramide synthesis
SMPD1	+	++	Sphingomyelin degradation
PLPP1	++	+	Dephosphorylation of sphingolipid intermediates
UGT8	+/-	++	Glucuronidation
KES1	+/-	+	Lipid transport

Fungal lineages adapted to aerobic environments (e.g., *Blastocladio-*, *Chytridio-*, *Zoopago-*, *Mortierello-*, *Mucoro-*, and *Glomeromycota*) possess a greater repertoire of sphingolipid (SL) metabolism proteins compared to microaerophilic or anaerobic taxa (*Neocallimastigo-*, *Rozellomycota*, and Microsporidia), regardless of the genome/proteome size (Fig. 1).

### *De novo* ceramide synthesis is preserved across fungi

**Figure 2.**
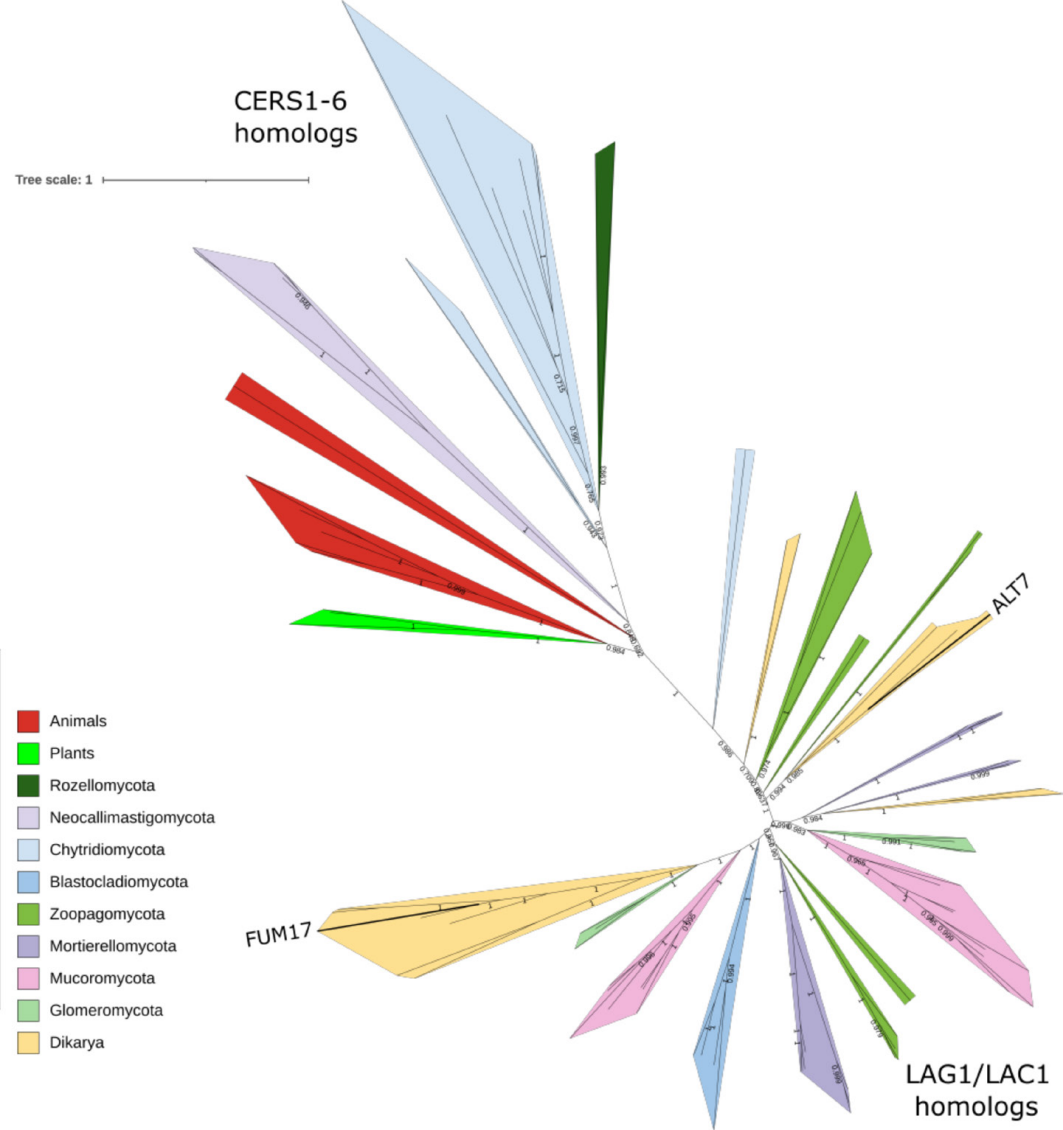
CERS1–6 form a separate clade distinct from typical fungal ceramide synthases. A maximum likelihood phylogenetic tree was computed with IQ-TREE 2.0.3 automated model selection (LG+R6) for 95 protein sequences with a TRAM_LAG1_CLN8 (PF03798) Pfam domain. Numbers on branches indicate bootstrap values above 0.5. Sphingosine *N*-acyltransferase-like proteins involved in the biosynthesis of mycotoxins FUM17 and ALT7 belong to two distinct fungal clades.

Most subfamilies involved in *de novo* ceramide biosynthesis are conserved across fungal genomes, with the exception of the *Saccharomycotina*-specific LIP1 and sphingolipid delta(4)-desaturase DES1. The latter is primarily restricted to *Dikarya* and only sporadically present in other fungal lineages (Fig. 1). The key enzyme in ceramide biosynthesis—the ceramide synthase family—is not only conserved across fungi but also has undergone multiple independent expansions in vertebrates and distinct fungal phyla, including *Mortierellomycota*, filamentous *Dikarya*, and *Entomophthoromycota* (Fig. 2) and *Basidiobolus meristosporus* (Suppl. material 1). Ceramide synthases can be phylogenetically divided into an animal-like clade, comprising all CERS1–6 reference sequences from animals, plants, and flagellated fungi, as well as distinct fungal-specific clades, which include the *Saccharomyces cerevisiae* ohnologs LAG1 and LAC1. These subfamilies transfer an acyl chain from acyl-CoA to a sphingoid base with varying substrate specificity, producing either dihydroceramide or ceramide. Some paralogs have acquired novel functions, such as sphingosine *N*-acyltransferase-like (ex., *Gibberella moniliformis* FUM17, W7LKY5; *Alternaria alternata* ALT7, G1UJF5), which are implicated in toxin biosynthesis (Fig. 2).

The final step of the *de novo* ceramide biosynthesis pathway is catalyzed by the desaturases: the above-mentioned sphingolipid delta(4)-desaturase DES1 (DEGS1) and sphingolipid delta(4)-desaturase/C4-monooxygenase DES2 (DEGS2) (Hannun and Obeid 2017). DEGS2 is more broadly distributed across early diverging fungal lineages than DEGS1 (Fig. 1), with the exception of *Neocallimastigomycota* and Microsporidia, and is not present in *Dikarya*. Substrate specificity differentiates the two enzymes: DEGS1 acts on sphinganine, whereas DEGS2 targets dihydroceramide, a central intermediate in *de novo* ceramide synthesis, which likely explains the widespread occurrence of DEGS2.

In addition to DEGS1 and DEGS2, several other desaturases involved in ceramide recycling or related pathways, such as acyl-CoA 6-desaturase, acyl-CoA desaturase/delta 9-fatty acid desaturase, and sterol desaturase, are also conserved and shared with animals. This, together with the conservation of DEGS1/2 in both fungi and animals, suggests these components represent ancestral traits of eukaryotic lipid metabolism (Suppl. material 1).

Notably, desaturases are absent from the genomes of anaerobic gut fungi (*Neocallimastigomycota*) and parasitic lineages such as *Rozellomycota* and Microsporidia. In contrast, several aerobic lineages, regardless of their lifestyle or ecological niche, encode up to 6–7 copies of acyl-CoA desaturase/delta 9-fatty acid desaturase. This expansion is observed in larval gut symbionts such as *Smittium megazygosporum* and *S. simulii*, the parasite *Entomophthora muscae*, as well as in motile saprotrophic chytrids like *Rhizoclosmatium globosum*, consistent with broader gene family expansions across the latter two genomes (Fig. 1).

### Blastocladiomycota bridge the diversity of sphingolipid recycling between flagellated and non-flagellated fungi

Once formed via the *de novo* pathway, ceramide can be either utilized directly or recycled into complex sphingolipids, such as sphingomyelin, sphingosine, glucosylceramide, and ceramide-1-phosphate. Several protein families involved in sphingolipid recycling, such as acyl-CoA desaturase/delta 9-fatty acid desaturase, sphingomyelin synthase, acid and neutral sphingomyelin phosphodiesterases, alkaline ceramidase, and phospholipid phosphatase, show notable expansions in non-flagellated fungi and are conserved across opisthokonts (Fig. 1). However, motile *Blastocladiomycota* retain ancestral fungal traits while also displaying evolutionary innovations typical of *Dikarya*, suggesting that complex sphingolipid metabolism may represent an adaptation linked to the transition to terrestrial environments.

### Sphingomyelin biosynthesis is conserved in non-Dikarya fungi, whereas its degradation pathways exhibit greater evolutionary diversification

Sphingomyelin synthase (SMS), along with acidic (ASMase) and neutral (NSMase) sphingomyelin phosphodiesterases characterized in animals, are key enzymes in sphingomyelin metabolism, a pathway essential for membrane homeostasis and potentially involved in terrestrial adaptation, yet it remains understudied in fungi. SMS catalyzes sphingomyelin synthesis from ceramide and phosphatidylcholine, while ASMase and NSMase mediate its degradation. SMS homologs are specific for non-*Dikarya* fungi and underwent lineage-specific duplications in *Allomyces macrogynus* (*Blastocladiomycota*) and *Entomophthora muscae* (*Entomophthoromycota*). SMS phylogeny reveals a distinct clade of animal SMS1/2 homologs and independent duplication events in fungi, with two separate clades observed in *Blastocladiomycota*, suggesting functional divergence (Fig. 1, Suppl. material 2).

**Figure 3.**
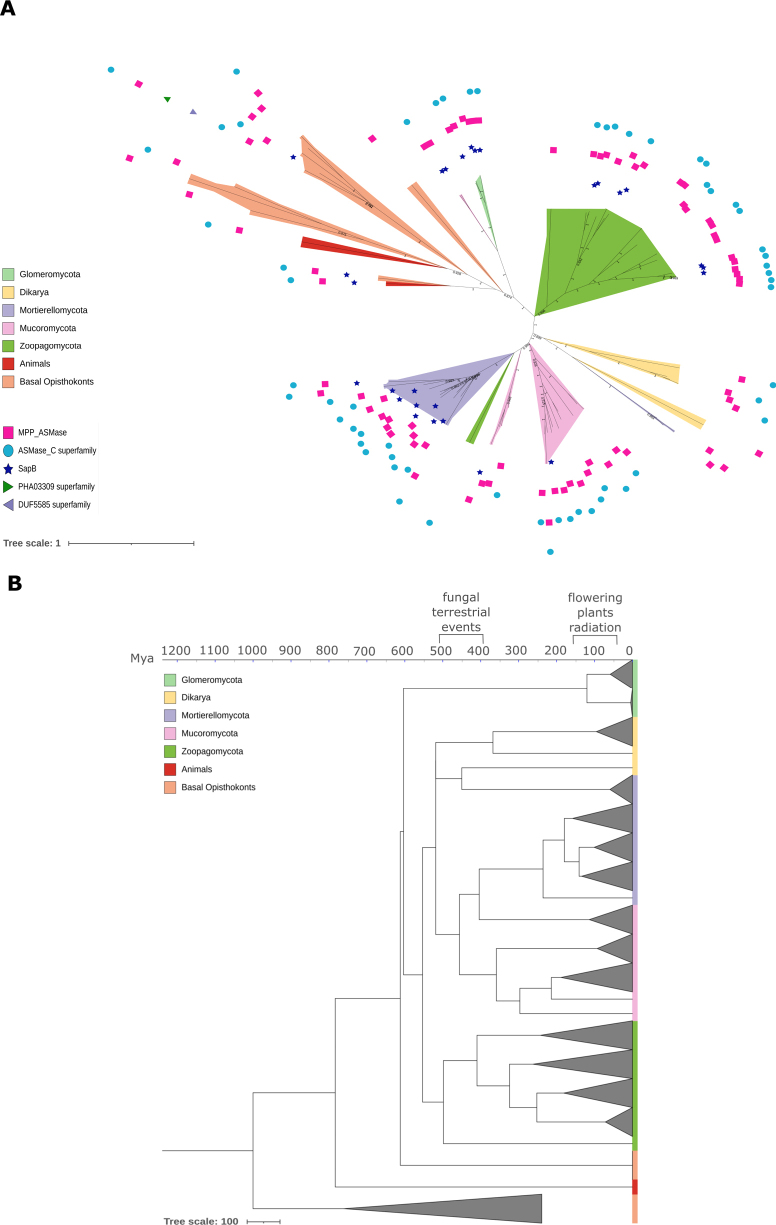
Phylogenetic tree of the acid sphingomyelin phosphodiesterase shows consecutive duplications. A maximum likelihood phylogenetic tree and a time-calibrated tree were computed with IQ-TREE 2.0.3 automated model selection (LG+R5) for 80 protein sequences with an ASMase_C (PF19272) Pfam domain. **A** Acid sphingomyelin phosphodiesterases show varying domain architecture across selected eukaryotic lineages. Numbers on branches indicate bootstrap values above 0.5; **B**ML-calibrated phylogenetic trees of the acid sphingomyelin phosphodiesterase protein family across selected eukaryotic lineages show consecutive duplications. The timing of fungal terrestrialization events is shown according to Liu et al. (2024) and Berbee et al. (2020), whereas the radiation of flowering plants is shown according to Dimitrov et al. (2023).

NSMase sequences form two main clades: ISC1 homologs (SMPD2-like, involved in inositol phosphorylceramide degradation), restricted to non-flagellated fungi, and SMPD3-like homologs (sphingomyelin degradation in the plasma membrane), which are ubiquitous across fungal lineages and animals but are particularly expanded in *Allomyces macrogynus* (*Blastocladiomycota*) (Suppl. material 2). Notable expansions of ASMase genes are observed in *Zoopago-* and *Mortierellomycota* and several *Dikarya* lineages, including *Yarrowia lipolytica* (which is a known oleaginous fungus), *Dacryopinax primogenitus*, and *Ramaria rubella*. Most fungal ASMases possess the conserved MPP_ASMase domain (CDD: cd00842), partially overlapping with the C-terminal ASMase_C domain (PF19272). Additionally, sequences from *Mortierello-* and some representatives of *Zoopago-*, *Glomero-*, and *Mucoromycota* contain an N-terminal Saposin-B domain (CDD: SapB, IPR008139), known from lysosomal proteins (Fig. 3A). Molecular dating indicates multiple lineage-specific ASMase expansions between ~200 and 300 Mya in *Zoopago-*, ~100–250 Mya in *Mortierello-* and *Mucoromycota*, as well as younger than 100 Mya in *Glomeromycota* and *Dikarya* (Fig. 3B). The most recent common ancestors (MRCAs) of these duplications seem to be overestimated and date back to ~430 Mya in *Zoopago-* and *Mortierello-*, ~380 Mya in *Mucoromycota*, which is close to the period of fungal land colonization events (Liu et al. 2024), as well as ~120 Mya in *Glomeromycota* and *Dikarya*, coinciding with the diversification bursts in the evolution of flowering plants (Dimitrov et al. 2023).

These results suggest that non-flagellated fungi possess enzymatic capacity to hydrolyze diverse complex sphingolipids, including inositol phosphorylceramide. Moreover, sphingomyelin degradation via sphingomyelinases is evolutionarily conserved and may represent an adaptation to terrestrial life, as particularly ASMases are restricted to non-flagellated fungi and are absent in *Blastocladiomycota*.

### Functional diversification of fungal ceramidases and DGK kinases reflects lineage-specific substrate preferences

Ceramidases, which hydrolyze ceramides into sphingosine, vary in optimal pH and subcellular localization. In fungi, we identified three types of ceramidases: acidic (Asah1, lysosomal), neutral (Asah2, plasma membrane), and alkaline (Asah3, ER/Golgi).

Interestingly, Asah1 homologs, typically associated with lysosomes, are mainly present in pathogenic *Pezizomycotina* and *Mortierellomycota*. Despite fungi lacking classical lysosomes, this suggests functional analogs or alternative localization. Asah2 is mostly restricted to *Chytridiomycota* and is rare among non-flagellated fungi (Fig. 1; Suppl. material 2).

Asah3 is the most broadly distributed and abundant, particularly enriched in *Mortierello-*, *Glomero-*, and *Blastocladiomycota*. The latter group and *Dikarya* cluster with animal alkaline ceramidase 3 (ACER3) homologs, which preferentially hydrolyze phytoceramides and canonical ceramides, such as *S. cerevisiae* Ypc1 and Ydc1. In contrast, *Mortierellomycota* branch exclusively with alkaline ceramidases 1 and 2 (ACER1/2), while other fungal lineages retain all three homologs (Suppl. material 2). ACER1/2 show substrate specificity for very long-chain saturated and short- to medium-chain ceramides, respectively, suggesting lineage-specific functional divergence in ceramide hydrolysis.

**Figure 4.**
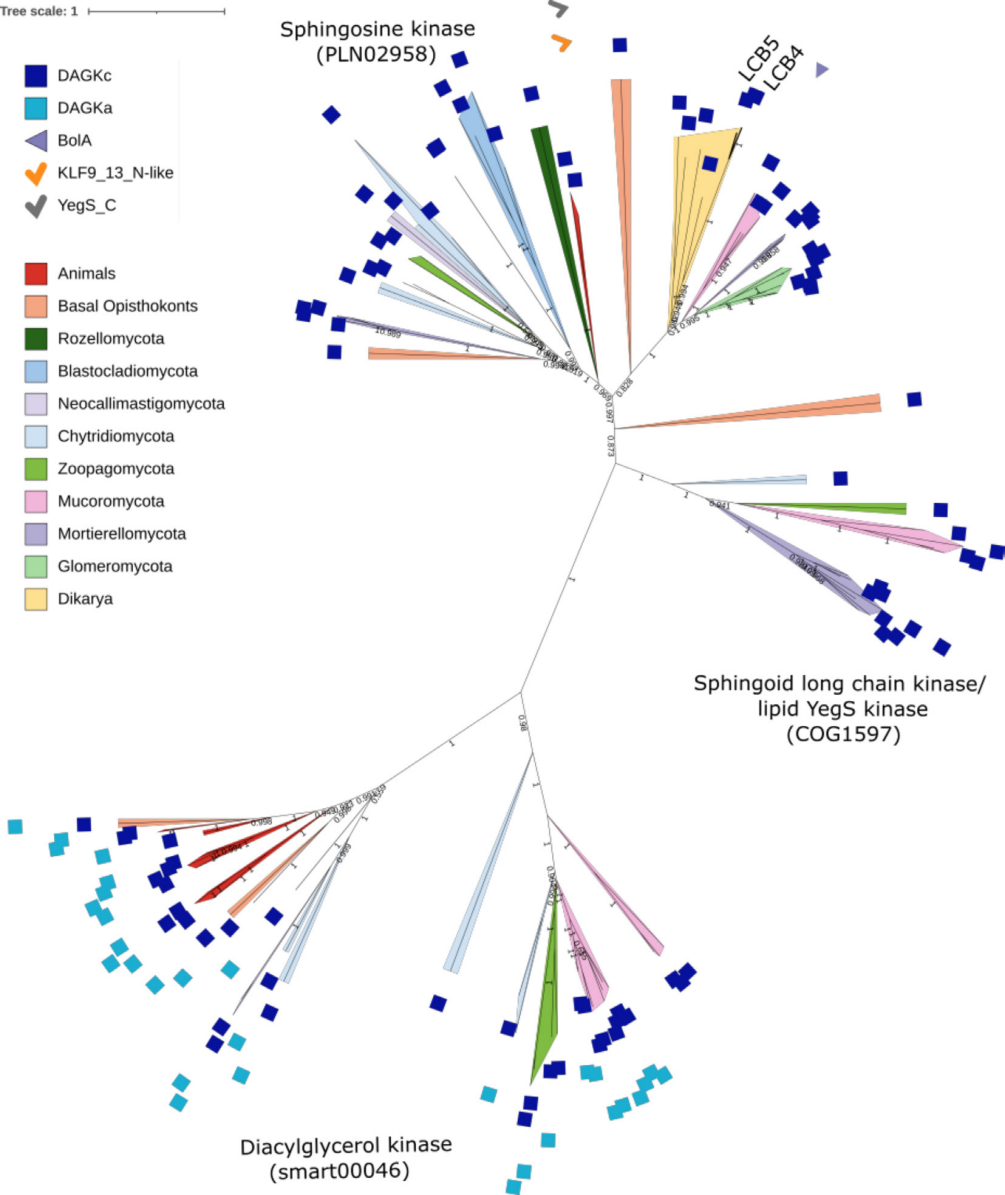
Diacylglycerol kinases often harbor additional domains and form distinct subfamilies. A maximum likelihood phylogenetic tree was computed with IQ-TREE 2.0.3 automated model selection (LG+R7) for 106 protein sequences with a DAGK_cat (PF00781) Pfam domain. Protein subfamilies are characterized by clade names. LCB4 and LCB5 (UniProt IDs: Q06147, Q12246, with Pfam domain PF00781) are *Saccharomyces cerevisiae* ohnologs encoding sphingoid long-chain base kinase (LCB). Numbers on branches indicate bootstrap values above 0.5.

Based on domain architecture, we identified three distinct DAGK-related kinase subfamilies in fungi: sphingosine kinase (SPHK), sphingoid long-chain base kinase (YegS-like), and diacylglycerol kinase (DGK) (Fig. 4). These subfamilies perform different enzymatic reactions.

SPHK, which catalyzes the phosphorylation of sphingosine to sphingosine-1-phosphate, is broadly conserved across fungi. Phylogenetic analysis reveals that flagellated fungi and *Zoopagomycota* cluster with animals, harboring an ancestral SPHK2-like enzyme. On the other hand, non-flagellated fungi possess homologs of the *S. cerevisiae* ohnologs LCB4 and LCB5 (UniProt Q06147, Q12246), which encode sphingoid long-chain base kinases that phosphorylate dihydrosphingosine and phytosphingosine to DHS-1P and PHS-1P, respectively. In *Spizellomyces punctatus* (*Chytridiomycota*, KND03613.1), *Dimargaris cristalligena* (*Zoopagomycota*), and *Mucoro-* and *Mortierellomycota* representatives, we found a YegS-like lipid kinase (COG1597, YegS_C domain, PF19279) of bacterial origin, most probably having a broader substrate range than other subfamilies. The YegS_C domain is a C-terminal extension found in a subset of lipid kinases containing the DAGK_cat domain (PF00781), including bacterial YegS as well as eukaryotic sphingosine and sphingoid base kinases (e.g., SPHK2, some fungal LCBs).

The ATP-dependent diacylglycerol kinase (DGK) catalyzes the conversion of diacylglycerol into phosphatidic acid and contains, in addition to the N-terminal DAGKc domain, a C-terminal DAGKa domain. DGKs are present in flagellated lineages and *Zoopagomycota* and underwent lineage-specific expansion in *Mucoromycota* but are absent from *Dikarya*. DGK duplications in *Mucorales* are estimated to have started ~200 Mya (Suppl. material 2). Phylogenetic analyses suggest that flagellated fungi retain an ancestral form of DGK, as their sequences cluster with those from animals and basal opisthokonts (Fig. 4). The observed patterns among DAGK kinases suggest functional diversification also within subfamilies.

### Sugars matter—the ability to decorate sphingolipids with glycosylations differs among fungi

Glycosyltransferases (GT) modify sphingolipids through lineage-specific reactions, including mannosyl-/inositol-, galactosyl-, and glucosylceramide synthesis. The most common GT families involved in sphingolipid glycosylation are GT1 (UGT), GT28, GT31, GT32, and GT33 families (Warnecke and Heinz 2003). For instance, we identified three GT32 subfamilies restricted to flagellated fungi and *Dikarya*, which are not shared with animals: alpha-mannosyltransferase (OCH1), which transfers mannose to oligosaccharides and is not directly involved in sphingolipid metabolism, mannosyl-phosphorylinositol ceramide synthase (SUR1), which catalyzes mannosylation of phosphorylinositol ceramide, and unannotated OCH1-like homologs found in *Aspergillus nidulans* and *Chytridiomycota*. The latter group and *Dikarya* possess all three GT32 subfamilies, whereas *Blastocladiomycota* and *Neocallimastigomycota* encode only alpha-mannosyltransferase. The highest copy number (5) was found in *Batrachochytrium dendrobatidis*, all belonging to the OCH1-like type. In contrast to alpha-mannosyltransferase, beta-1,3-galactosyltransferase 6, belonging to GT31, is absent in flagellated fungi, *Dikarya*, and *Zoopagomycota* but present in other non-flagellated fungal lineages and animals, suggesting that galactosylation is evolutionarily older than mannosylation (Fig. 1, Suppl. material 2).

**Figure 5.**
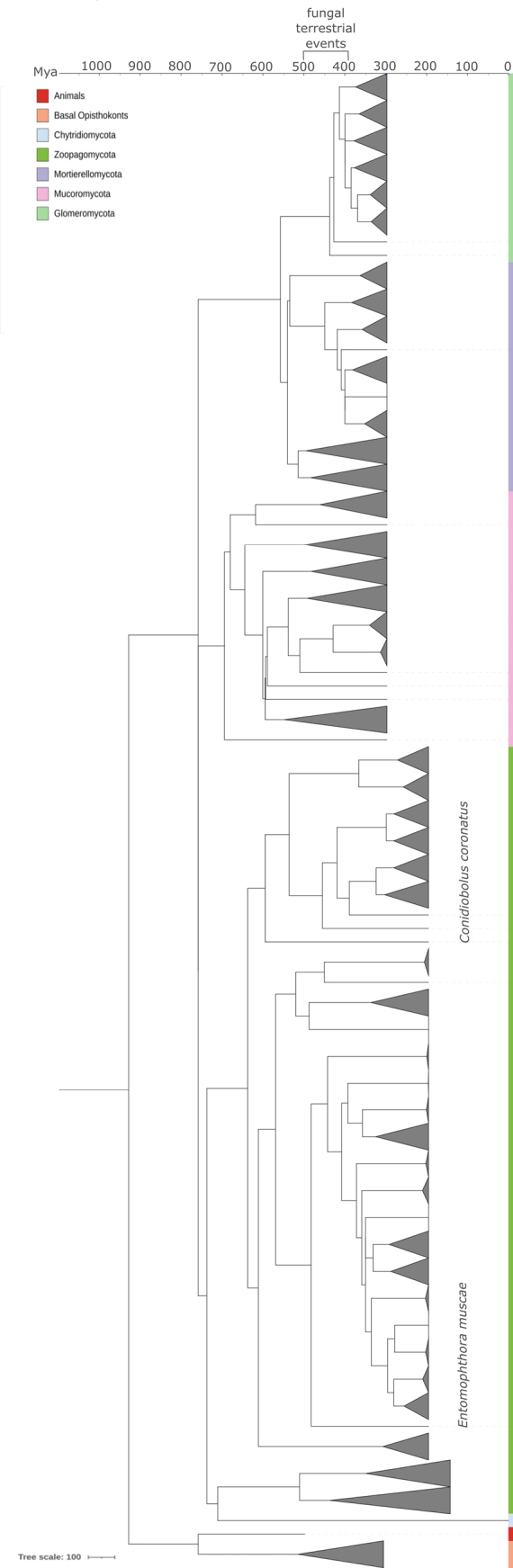
Calibrated phylogenetic tree of UGT8-like UDP-glucuronosyltransferase shows consecutive duplications. A maximum likelihood phylogenetic time-calibrated tree was computed with IQ-TREE 2.0.3 automated model selection (LG+F+R10) for 188 protein sequences with a UDPGT (PF00201) Pfam domain. Fungal terrestrialization events are shown according to Liu et al. (2024) and Berbee et al. (2020 ).

Multiple copies of UGT8-like UDP-glycosyltransferases (GT1) were identified in *Zoopago-*, *Mucoro-*, and *Glomeromycota*, potentially exhibiting both UDP-glucuronosyltransferase and *N*-acylsphingosine galactosyltransferase activity—the latter shared with animals but absent in flagellated fungi. These duplications are estimated to have occurred around ~200–300 Mya in *Conidiobolus coronatus* and *Entomophthora muscae*. The diversification of glucuronyltransferases in non-flagellated lineages suggests a selective role of glucuronidation in membrane specialization outside aquatic environments (Fig. 5). A contrasting pattern was observed for sterol 3-beta-glucosyltransferase (ATG26; GT28), which glycosylates ergosterol and other membrane sterols. ATG26 homologs are widespread across fungi and frequently duplicated, with six paralogs identified in *Puccinia graminis*, but are absent in animals (Fig. 1).

### Simple lipid metabolism in fungi: evolutionary links with animals and plants

Ceramide metabolism depends on the availability of simple lipids such as diacyl-, triacylglycerol, and waxes. We identified four protein subfamilies involved in their metabolism across opisthokonts: phospholipid phosphatase (PLPP1) and three acyltransferases—DGAT1/SOAT1, DGAT2, and WSD1 (Fig. 1). DGAT1 and SOAT1 belong to two families of acyltransferases described in animals differing in substrate specificity (diacylglycerol vs. sterol), while WSD1 is more common in plants but also present in some invertebrates (ex., *Drosophila*). DGAT1/SOAT1, DGAT2, and PLPP1 are widely conserved in fungi, with lineage-specific expansions of DGAT2 and PLPP1, especially in *Zoopagomycota*. WSD1 is less frequent, but remarkably expanded in *Gonapodya prolifera* (>5 copies). In *Neocallimastigomycota*, despite a reduced lipid metabolic repertoire, PLPP1 is duplicated, possibly compensating for other lipid pathway reductions by enhancing diacylglycerol availability for membrane remodeling in anaerobic niches such as the herbivore gut.

### Evolutionary innovations in fungal sphingolipid metabolism and conserved ceramide and lipid crossroads

**Figure 6.**
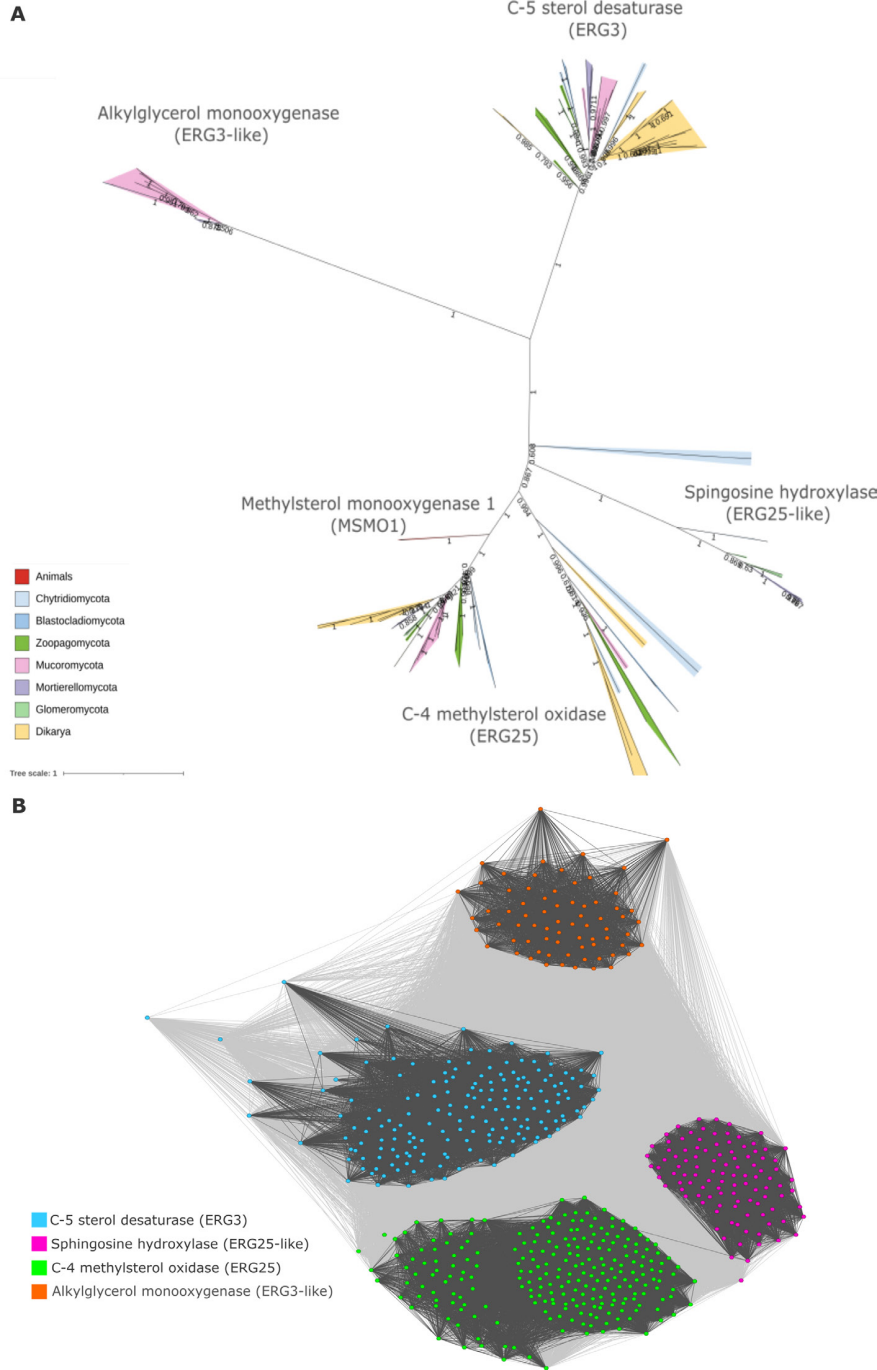
Sterol desaturases form fungal-specific subfamilies. A maximum likelihood phylogenetic tree was computed with IQ-TREE 2.0.3 automated model selection (LG+I+G4) for 118 protein sequences with a FA_hydroxylase (PF04116) Pfam domain. Numbers on branches indicate bootstrap values above 0.5. **A** Four sterol desaturase protein subfamilies: C-4 methylsterol oxidase (ERG25; homolog of animal methylsterol monooxygenase—MSMO1), sphingosine hydroxylase (ERG25-like), C-5 sterol desaturase (ERG3), and alkylglycerol monooxygenase (ERG3-like); **B** Four distinct clusters identified by CLANS among sterol desaturase family sequences.

Out of the 59 analyzed proteins, 16 catalyze fungal-specific reactions, with five—IPT1, ATG26, OSH2/3, and ERG3/25—conserved across fungal lineages, pointing to inositol phosphate transfer to ceramides, sterol gly cosylation, lipid transport, and sterol biosynthesis as core traits of sphingo-/lipid metabolism (Fig. 6). The fungal-specific rare enzymes include a YegS-like lipid kinase, dihydrosphingosine 1-phosphate phosphatase, three sphingolipid C9-methyltransferases (MTS, MT1, MT2), mannosyltransferases, and wax ester synthase (Fig. 1).

MTS and MT1/2 are specific to *Blastocladiomycota*, whereas *Neocallimastigo-* and *Mortierellomycota* lack them almost entirely. These paralogous enzymes catalyze fungal-specific glucosylceramide methylation at the C-9 position of the sphingoid base, impacting membrane organization and host interactions. While present in several other fungal lineages, MTS is the rarest among these enzymes.

Most enzymes involved in both ceramide and broader lipid metabolism are conserved within opisthokonts. However, some display pronounced lineage-specific diversity, including very long-chain fatty acid elongases (ELOVL), oxysterol-binding proteins, and lipid transporters such as OSH1/2 and KES1 (Fig. 1). We identified four ELOVL subfamilies, suggesting differences in substrate specificity (Suppl. material 2). Gene duplications within a conserved ELOVL subfamily occurred in *Entomophthora muscae* (*Zoopagomycota*) and *Neocallimastix californiae* (*Neocallimastigomycota*), while a delta6-elongase subfamily was specific to *Glomeromycota* and *Mortierellomycota*. KES1 is restricted to non-flagellated fungi, except for *Blastocladiomycota*, where its presence may reflect intermediate traits between aquatic and terrestrial fungi.

### Genes linked to *de novo* and recycling ceramide pathways are expressed in fungi

**Figure 7.**
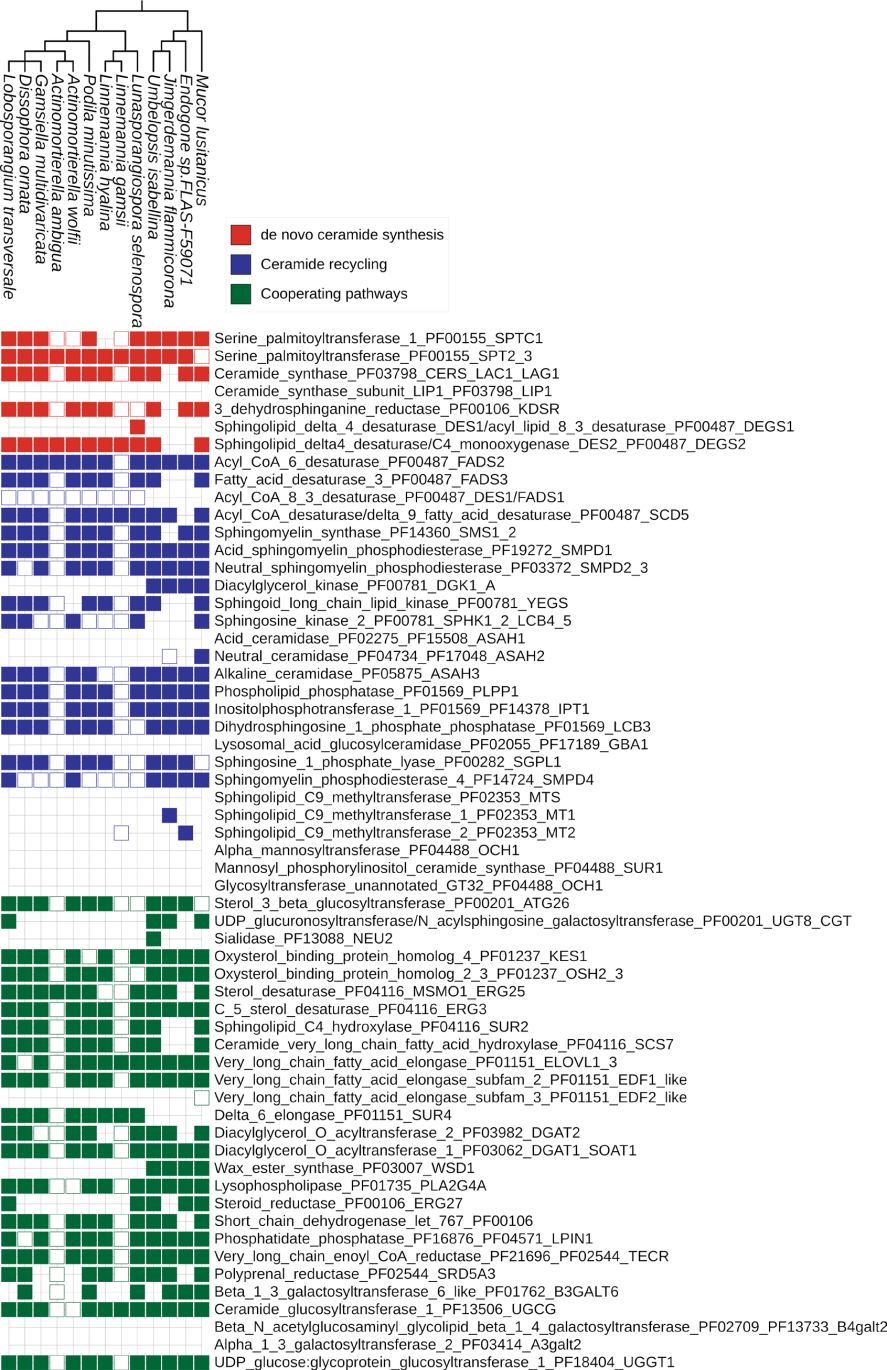
Genes encoding sphingolipid metabolism enzymes are active across diverse fungal representatives. Identification of transcripts was performed for all sphingolipid metabolism genes in a given species in public datasets from the axenic culture transcriptomes of *Mucoromycota* and *Mortierellomycota*. The filled squares represent the expression of the gene encoding a given protein; the colored squares represent only the presence of a given gene.

**Figure 8.**
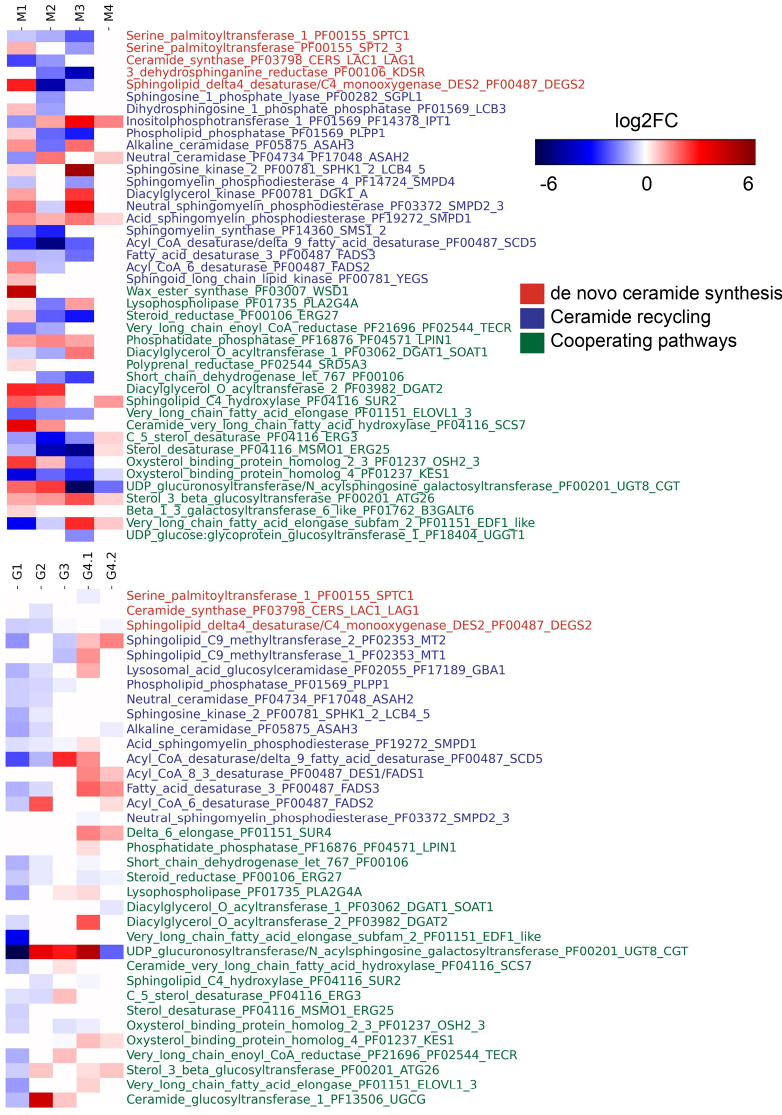
Genes encoding sphingolipid metabolism enzymes are differentially expressed. Differential expression of sphingolipid metabolism genes was analyzed for public RNA-Seq datasets storing information on gene expression under diverse environmental conditions in selected members of *Mucoromycota* (M) and *Glomeromycota* (G). **M1**: *Mucor lusitanicus* growth in anaerobic vs. aerobic conditions; **M2**: *Rhizopus delemar* growth in the presence vs. absence of murine macrophages; **M3**: *Rhizopus delemar* host–pathogen interaction (18 h) using mouse bone marrow-derived macrophages (BM); **M4**: *Rhizopus delemar* host–pathogen interaction (16 h) using human airway epithelial cells (A549); **G1**: *Rhizophagus irregularis* mycelial growth in association with *Lotus japonicus*; **G2**: *Rhizophagus irregularis* association with *Medicago truncatula* and treatment with strigolactone for 24 h; **G3**: *Gigaspora margarita* growth in association with *Candidatus Glomeribacter gigasporum*; **G4.1**: *Gigaspora rosea* response to plant signals in the switch from asymbiotic to presymbiotic growth in the presence of *Daucus carotta* roots; **G4.2**: *Gigaspora rosea* response to plant signals in the switch from asymbiotic to presymbiotic growth in the presence of GR24 (synthetic analog of strigolactone).

Available transcriptomes of axenic cultures of *Mucoro-* and *Mortierellomycota* representatives show the expression of a number of predicted SL metabolism-related genes (Fig. 7). The patterns of gene expression differ between species and environmental conditions (Fig. 8). For example, *Linnemannia gamsii* and *Actinomortierella ambigua* datasets show expression of 6/40 and 4/40 SL genes, respectively. On the other hand, the datasets for *Lobosporangium transversale*, *Endogone* sp., and *Umbelopsis isabellina* present expression of almost all analyzed SL genes (40/41, 31/31, and 41/41, respectively) (Fig. 7). We observe expression of at least four genes encoding SL-related proteins in all of the 13 available and analyzed axenic culture transcriptomes of *Mucoro-* and *Mortierellomycota* (Fig. 7). The expression of predicted genes is generally similar for both *Mucoro-* and *Mortierellomycota*, with a few exceptions described below. All four available pure transcriptomes of *Mucoromycota* express most of the proteins of SL metabolic pathways. For instance, in *de novo* ceramide synthesis, only the serine palmitoyltransferase gene (SPT2/3) in *M. lusitanicus* is not expressed. The rest of the genes are expressed, including the housekeeping ceramide synthase (CERS/LAC1/LAG1). On the other hand, the selected nine transcriptomes of *Mortierellomycota* show scattered expression patterns for the same. In ceramide recycling, key genes encoding enzymes of sphingomyelin metabolism, such as sphingomyelin synthase (SMS1/2) and acidic and neutral sphingomyelin phosphodiesterases (SMPD1 and SMPD2/3, respectively), are expressed in all species except *L. gamsii* and *A. ambigua*. Likewise, genes encoding enzymes involved in synthesis of complex sphingolipids in cooperating pathways, such as UDP-glucuronosyltransferase (UGT8/CGT) or oxysterol-binding protein homologs (KES1 and OSH2/3), are expressed in all selected *Mucoromycota*; however, they have uneven patterns of expression in *Mortierellomycota*.

When it comes to the expression profiling of the predicted genes in condition-specific transcriptome analysis, a greater number of genes are differentially expressed in *Mucoro-* than in *Glomeromycota* (Fig. 8). In both groups, genes involved in *de novo* ceramide synthesis are either downregulated or not expressed at all, whereas genes in ceramide recycling and associated pathways exhibit species- and condition-specific patterns. For instance, *M. lusitanicus* under anaerobic conditions (Fig. 8, **M1**) shows the highest number of differentially expressed genes (38/42) in ceramide metabolism, predominantly from recycling pathways and mostly upregulated (23/38). By contrast, in *R. irregularis* during mycelial growth with *Lotus japonicus*, genes are either silent or downregulated (Fig. 8, **G1**). Association with *Medicago truncatula* and short-term strigolactone treatment reverses this pattern, leading to upregulation of acyl-CoA 6-desaturase, sterol 3-beta-glucosyltransferase, UDP-glucuronosyltransferase, and ceramide glucosyltransferase 1. A clear difference is seen when *G. margarita* in association with *Candidatus Glomeribacter gigasporarum* (**G3**) is compared to *R. irregularis* with plants (**G1**), as acyl-CoA/delta-9 desaturase 2 and C-5 sterol desaturase are upregulated only in the bacterial–fungal association. Finally, in *R. delemar* grown in A549 human airway epithelial cells (**M3**), only a subset of genes from ceramide recycling and cooperating pathways is expressed, the majority being upregulated. Genes with high copy numbers (e.g., UGT8/CGT, DEGS2, SMPD1, ASAH3, OSH2–3, KES1, and ELOVL1–3) show diverse expression patterns across transcriptomes. For instance, UGT8/CGT shows elevated expression only in the M1, M2, G2, G3, and G4.1 transcriptomes while being downregulated in the remaining datasets.

## Discussion

Sphingolipids are essential membrane components and signaling molecules. Disruption of *de novo* ceramide synthesis causes growth and sporulation defects in yeasts (Buede et al. 1991) and other *Dikarya*, such as *Aspergillus nidulans* and *Neurospora crassa* (Li et al. 2006; Huber et al. 2019). Current knowledge of fungal sphingolipid metabolism is largely derived from *Dikarya*, and phenotypic data on sphingolipid gene deletions or mutations outside this clade remain scarce (Usmani et al. 2023). In fungi, particularly within *Saccharomycotina*, expansions of gene families have been linked to enhanced metabolic versatility (David et al. 2024). Our findings extend this observation to sphingolipid metabolism, highlighting that gene family evolution is associated with the diversification of sphingolipid repertoires across fungi. The conservation of a subset of sphingolipid-related proteins across major fungal phyla supports their essential, housekeeping roles. In contrast, the presence of lineage-specific proteins points to evolutionary innovation, suggesting that distinct fungal groups may have developed unique sphingolipid metabolic strategies to adapt to their ecological niches. Interestingly, we found that only a few subfamilies could have a history of non-vertical descent (YEGS and secondary metabolite clusters with ceramide synthase genes). Of the 59 protein families analyzed, most are shared with other opisthokonts, consistent with an ancestral origin, whereas distinct subsets are restricted to either *Dikarya* and non-*Dikarya* or to flagellated and non-flagellated fungi, indicating lineage-specific adaptations.

In eukaryotes, enzymes of *de novo* sphingolipid synthesis and recycling are generally conserved (Harrison et al. 2018). However, fungal SL are chemically distinct from those of plants and mammals in their sphingoid bases, α-hydroxylated fatty acids, and head group modifications, reflecting variation in SL profiles, which possibly correlates with organismal lifestyle and specialized functions (Usmani et al. 2023; Jamjoum et al. 2024). We found that five of seven *de novo* components are conserved across fungi. For instance, serine palmitoyltransferase is retained in most fungal genomes, even in Microsporidia, which preserve only ~14.5% of sphingolipid-related protein families (Fig. 1, Suppl. material 1). These parasitic organisms are obligate anaerobes that rely on host-derived fatty acids (Biderre et al. 2000; Nev et al. 2024), and our results extend the list of known pathway losses in this lineage.

Notably, most desaturases involved in sphingolipid and fatty acid metabolism are absent from motile fungi (except *Blastocladiomycota*) and from parasitic Microsporidia. For instance, sphingolipid delta(4)-desaturase/C4-monooxygenase DES2 (DEGS2) is broadly conserved across non-flagellated fungi, together with acyl-CoA 6-desaturase (FADS2) and acyl-CoA desaturase/delta-9 desaturase 2 (SCD5) (Fig. 1). Available expression data indicate complementary functions of these desaturases: FADS2 is primarily associated with lipid/membrane homeostasis, whereas SCD5 shows condition-dependent regulation during host–pathogen interactions. SCD5 introduces double bonds in palmitoyl (16:1) and linolenic (18:1) fatty acids, which can be inserted into ceramides by ceramide synthase, contributing to their accumulation, and work as bioactive lipids in fungal pathogenesis (Ali et al. 2024). Desaturase diversity has also been linked to oleaginous traits, particularly in *Mucorales* (Aguilar and De Mendoza 2006). *Mucoro-*, together with *Mortierello-* and *Ascomycota*, encodes multiple desaturases, including delta-6 (FADS2), delta-5 (FADS1), delta-15 (DEGS1), delta-12 (FADS12), and delta-9 (SCD5) (Vorapreeda et al. 2020; Sokołowska et al. 2023). Similarly, our data show that other desaturases such as FADS3 and DEGS2 are also largely restricted to *Mucoromycota* (Suppl. material 1).

By contrast with the universally conserved *de novo* pathway, proteins involved in ceramide recycling and associated pathways are more abundant in non-motile lineages. As most ceramide recycling genes are shared with opisthokonts (20/22; Suppl. material 1), the expanded repertoire of enzymes involved in ceramide recycling may represent either the ancestral pathway or a version favored in terrestrial environments. On the other hand, anaerobic fungi—including obligate anaerobes and microaerophilic symbionts or parasites such as *Neocallimastigo-*, *Rozellomycota*, and Microsporidia—show a pronounced reduction in SL gene repertoires (Fig. 1, Suppl. material 1), likely reflecting adaptation to their specialized ecological niches.

Molecular dating indicates successive duplications of ceramide recycling genes started ~200–300 Mya (e.g., acid sphingomyelin phosphodiesterase, UDP-glucuronosyltransferase, or diacylglycerol kinase; Figs 3, 5), close to the estimated timeline of land colonization (~400–500 Mya), exemplified by Rhynie chert (Scotland, ~410 Mya) (Berbee et al. 2020; Gan et al. 2021; Glick et al. 2024; Liu et al. 2024). These gene dating estimates are close to the MRCA of non-flagellated fungi and suggest early changes in SL metabolism composition in the evolution of terrestrial fungal lineages, with another later expansion in arbuscular mycorrhizal *Glomeromycota* (~120 Mya) overlapping with the diversification of flowering plants (Dimitrov et al. 2023).

This observation adds to the known transition from ancestral sterols like cholesterol, 24-methyl sterols, such as lanosterol, campesterol, or sitosterol, to the dominant ergosterol among *Dikarya* (Weete et al. 2010; Sokolov et al. 2024). Interestingly, *Kickxello-*, *Entomophthoro-*, *Mortierello-*, and *Glomeromycota* display a combination of ancestral features: sphingomyelin and no ergosterol despite the loss of the flagellum. Two other non-flagellated lineages, *Zoopago-* and *Mucoromycota*, possess a combination of the ancestral trait sphingomyelin metabolism and a derived state, ergosterol, as the main sterol. The third combination of two derived traits is present in *Dikarya*, which have lost sphingomyelin and have ergosterol as the dominant sterol. Ergosterol interacts with unsaturated fatty acids to enhance membrane fluidity, alter permeability, and modulate the activity of embedded enzymes (Aguilar and De Mendoza 2006; *Biophysical Approaches for the Study of Membrane Structure Part B* 2024). The lack of ergosterol in some of the non-flagellated phyla is explained by missing ERG2, ERG5, and ERG4 genes. For historical reasons, other genes in the sterol pathway are also labeled with ERG names; however, most of them are not directly involved in ergosterol synthesis. For instance, the universally conserved ERG3 and ERG27, regardless of the dominant sterol, are pivotal for the core of the sterol biosynthesis pathway. ERG3 contributes to desaturation steps common to both cholesterol and ergosterol production, while ERG27 functions within the conserved C-4 demethylation process. These enzymes most likely function as membrane remodeling agents across fungi.

While ERG genes are central to sterol-mediated membrane regulation, other enzymes, such as ceramidases, contribute significantly to the maintenance of ceramide levels. These enzymes hydrolyze dihydroceramides to generate ceramides, promoting the evolution of cellular compartments by redistributing ceramides to plasma membranes and lysosomes (Fabrias et al. 2012). In fungi, this process most likely involves alkaline ceramidase, the most widespread type (Fig. 1). By contrast, acid ceramidase, associated with lysosomal function and pathogenicity (Ishibashi et al. 2012; Ito et al. 2014), is rare, and we detected Asah1 specifically in pathogenic *Asco-* and *Basidiomycetes* (Fig. 1). This enzyme may represent a dikaryan evolutionary novelty, as the yeast vacuolar membrane (functionally analogous to lysosomes in fungi) contains low levels of sphingolipids and exhibits a reduced ergosterol-to-phospholipid ratio (Li et al. 2006).

In addition to gene losses in sphingolipid metabolism, we detected recent duplications in several isolates, particularly in the oleaginous yeast *Yarrowia lipolytica* (Fig. 1) and in the aerobic entomoparasite *Entomophthora muscae* (Figs 1, 2, 3, 4, 5). Gene duplications in fungi are proposed to be associated with ecological adaptation (Orłowska and Muszewska 2022; Stajich et al. 2024). For instance, *Y. lipolytica* carries multiple copies of acid sphingomyelin phosphodiesterase, which may be linked to its oleaginous phenotype (Jiang et al. 2021; Han et al. 2021). In animals, sphingomyelin is ubiquitous, acting as a reservoir of ceramides and influencing the melting temperature of cellular membranes, thereby contributing to temperature sensing (Zhu et al. 2023). Moreover, sphingomyelin can form more hydrogen bonds than ceramide, enhancing lipid bilayer stability (Futerman 2024). These features may have particular relevance for terrestrial adaptation, where temperatures are less stable than in aquatic habitats. In fungi, however, sphingomyelin has so far been identified only in *Umbelopsis isabellina* (*Mucoromycota*) (Bernat et al. 2018) and remains largely unexplored. It has been proposed that in fungi, sphingomyelin may primarily function in bacterial–fungal interactions (Matos et al. 2023). Whereas sphingomyelin biosynthetic processes appear evolutionarily conserved in non-*Dikarya* fungi, the corresponding degradation pathways show substantially greater diversification (Fig. 1). In line with Sokołowska et al. (2023), we found that Isc1p (a neutral sphingomyelin phosphodiesterase subfamily, SMPD2-like homologs) is present in *Dikarya* and *Mucoro-* but absent from *Blastocladiomycota*, which instead possess SMPD3-like homologs—paralogs of SMPD2 (Wang et al. 2024).

In rare cases, we identified co-occurring lineage-specific expansions, such as those observed in *E. muscae*. In this organism, several genes duplicated—including sphingomyelin synthase and acid sphingomyelin phosphodiesterase (SMPD1 homologs, Fig. 3), but also acyl-CoA desaturase, phospholipid phosphatase, oxysterol-binding protein homologs (OSH2/3), very long-chain fatty acid elongases, diacylglycerol *O*-acyltransferase 2, and UDP-glucuronosyltransferase. These genes are functionally linked to both *de novo* ceramide biosynthesis and recycling (Fig. 1). Interestingly, UDP-glucuronosyltransferase, which mediates glucosylceramide (GluCer) synthesis, has been associated with fungal virulence (Cummings 2023), potentially explaining its expansion in the entomopathogen *E. muscae*.

In the *Mucorales* representative *R. delemar*, GlcCer represents the second most abundant class of complex SL (Ali et al. 2024). During host–pathogen interactions, *de novo* ceramide synthesis is downregulated, whereas sphingomyelin catabolism is upregulated. The increased expression of acid and neutral sphingomyelin phosphodiesterases, alkaline ceramidase, diacylglycerol kinase, and sphingosine kinase 2, together with the decreased expression of UDP-glucuronosyltransferase, indicates a metabolic shift from complex sphingolipid biosynthesis toward ceramide recovery via the degradation of complex sphingolipids (Fig. 8B).

Ceramide synthase has recently been identified as a potential antifungal target (Dasilva et al. 2025), and its deletion in pathogenic fungi has been shown to abolish virulence (Flor-Parra et al. 2021; Zamith-Miranda et al. 2021). Furthermore, the downregulation of ceramide synthase genes in selected *Mucoromycota* in response to stress-related environments suggests a suppression of sphingolipid biosynthesis as part of their stress adaptation (Fig. 8: **M1**; **M2**). Ceramide synthases seem to contribute in at least two parallel ways to fungal virulence, by stress adaptation and as part of secondary metabolite clusters (Krska et al. 2024). Interestingly, paralogs involved in secondary metabolism are closely related to the housekeeping enzymes and seem to have originated several times during fungal evolution, since sequences from *Alternaria alternata* (ALT7, LAG1 homologs) and *Gibberella moniliformis* (FUM17, FUM18, LAC1 homologs) group within the LAC1 and LAG1 clusters (Fig. 2).

The maintenance of membrane lipid homeostasis is also facilitated by diacylglycerol kinase (DGK), which is crucial under fatty acid limitation (Ma et al. 2019). When fatty acids are in excess, acyltransferases recycle them into bioactive sphingolipid species (Zhang et al. 2018). DGK activity is CTP-dependent in *Dikarya* and ATP-dependent in non-*Dikarya* fungi (Sokołowska et al. 2023). The latter enzyme underwent an expansion within *Mucoromycota* (~200 Mya) and may compensate for missing ceramide kinase (CerK) that, in animals, phosphorylates ceramide to ceramide-1-phosphate (Yamazaki et al. 2023). This functional overlap suggests that fungal enzymes can evolve alternative functions to maintain lipid homeostasis. In contrast to the diverse convergent and divergent diacylglycerol kinases, the diacylglycerol *O*-acyltransferases 1 and 2 (DGAT1/SOAT1, DGAT2) are conserved across fungi (Fig. 1). Moreover, DGAT2 is described as an ancient ‘paleo-protein’ crucial for terrestrial adaptation due to its high efficiency in low-oxygen conditions compared to DGAT1, making it well suited for early land colonizers in semi-aquatic or hypoxic niches.

A major challenge in genome-scale analysis is the limited availability of transcriptomic datasets for non-model taxa. In the analyzed transcriptomes of non-*Dikarya*, most of the ceramide genes with high copy number were expressed (Fig. 8). In certain flagellated fungal lineages, OSH3, PLPP1, OCH1, SUR1, and SMS1–2 were particularly frequent, whereas in non-flagellated lineages—KES1, SMPD1, LAC1/LAG1, SPHK2, LCB4–5, and UGT8.

We observed that *de novo* ceramide synthesis genes are downregulated under stress conditions in the analyzed transcriptomic datasets, suggesting a shift in sphingolipid composition toward the salvage pathway under adverse conditions (Fig. 8, **M2–M4**, **G1–G4.2**). The recycling of ceramides is enhanced in the case of *Mucoro-* and selected *Glomeromycota*, as observed in the condition-specific expression (Fig. 8, **M1–M4**, **G1–G4.2**). Upregulated expression of DGK1, SPHK2, and CERS observed in *R. delemar* host–pathogen interactions (Fig. 8, M4, M5) suggests that these genes may play an adaptive role in pathogenicity. Analysis of differentially expressed genes in *R. delemar* suggests that non-flagellated fungi have a more complete sphingolipid recycling over the *de novo* synthesis pathway, reflecting a strategy to regulate intracellular ceramide levels, particularly during pathogenic processes and potentially in response to terrestrial environmental stresses.

## Conclusion

Our findings highlight the relationship between sphingolipid metabolism and the ecological and evolutionary diversification of fungi. Motile *Blastocladiomycota* may represent an intermediate stage in sphingolipid metabolism between flagellated and non-flagellated fungi. The variation in key metabolic enzymes reflects their diversity and indicates a possible role in adapting to terrestrial environments. Further investigation into these metabolic pathways across diverse taxonomic groups may provide valuable insights into the evolution of lipid homeostasis and its significance for organismal survival in a changing environment.

## Additional information

### Artificial Intelligence (AI) use

Regarding the use of AI in the preparation of this manuscript, the authors declare the following:

**Description**: ChatGPT (OpenAI) was used for English proofreading.

### Adherence to national and international regulations

All the fungal strains used in this study have been legally obtained, respecting the Convention on Biological Diversity (Rio Convention).

### Author ORCIDs

Magdalena Płecha https://orcid.org/0000-0002-8946-907X

Drishtee Barua https://orcid.org/0000-0002-5555-7047

Julia Bartczak https://orcid.org/0009-0004-5278-8431

Anna Muszewska https://orcid.org/0000-0002-4578-844X

### Data availability

Additional materials are available in the Zenodo repository: 10.5281/zenodo.17600982. Gene names, protein accessions, taxonomic distribution of protein families, expression levels of selected genes listed as TPM values, transcriptomic datasets analyzed, and identifiers of analyzed fungal genomic assemblies are listed in Suppl. material 1. Images of other phylogenetic ML trees and dated trees are shown in Suppl. material 2.

## Supplementary materials

Supplementary material 1 Accessions and identifiers Data type xlsx Explanation note Gene names, protein accessions, taxonomic distribution of protein families, expression levels of selected genes listed as TPM values, transcriptomic datasets analysed and identifiers of analysed fungal genomic assemblies. https://binary.pensoft.net/file/1623040This dataset is made available under the Open Database License (http://opendatacommons.org/licenses/odbl/1.0/). The Open Database License (ODbL) is a license agreement intended to allow users to freely share, modify, and use this Dataset while maintaining this same freedom for others, provided that the original source and author(s) are credited. Magdalena Płecha, Drishtee Barua, Anna Muszewska

Supplementary material 2 Images of other phylogenetic ML trees and dated trees Data type pdf https://binary.pensoft.net/file/1623041This dataset is made available under the Open Database License (http://opendatacommons.org/licenses/odbl/1.0/). The Open Database License (ODbL) is a license agreement intended to allow users to freely share, modify, and use this Dataset while maintaining this same freedom for others, provided that the original source and author(s) are credited. Magdalena Płecha, Anna Muszewska
