# Supplementary material for: Evolutionary diversity of sphingolipid metabolism proteins in fungi
Source: IMA Fungus. 2026 May 4;17:e177891. doi: 10.3897/imafungus.17.177891 (PMC13161914; doi:10.3897/imafungus.17.177891)

Evolutionary diversity of sphingolipid metabolism proteins in fungi

Magdalena Płecha[1], Blanka Sokołowska[1,3], Drishtee Barua[1,2], Julia Bartczak[1,4], Maria Sobczyk[1], Dagmara Koperska[1], Anna Kułakowska[1], Adam Komosa[1], Anna Muszewska[1]*

List of images of maximum likelihood phylogenetic trees as well as dated trees across selected eukaryotic lineages inferred with IQTREE2 and rendered with IToL; numbers on branches indicate bootstrap values above 0.5. Dating and calibration of the time trees were performed with the LEAST SQUARE DATING (LSD2) method applied in IQ-TREE 2.0.3.

Fatty acids metabolism **2**
Desaturases 2
Acyl-CoA 6-desaturase (PF00487, FADS2) 2
Acyl CoA-desaturase/delta-9 fatty acid desaturase (PF00487, SCD5) 3
Ceramide biosynthesis **4**
Serine palmitoyltransferase (PF00155, SPT2/3) 4
Sphingomyelin synthase (PF14360, SMS) 5
Elongases 6
Very long chain fatty acid elongases (PF01151, ELOVL) 6
Acyltransferases 7
Diacylglycerol O-acyltransferase 2 (PF03982, DGAT2) 7
Diacylglycerol O-acyltransferase 1 (PF03062, DGAT1/SOAT1) 8
Wax ester synthase (PF03007, WSD1) 9
Lipases 10
Lysophospholipase (PF01735, PLA2G4A) 10
Lipids transfer **11**
Oxysterol-binding protein homolog 4 (PF01237, KES1) 11
Oxysterol-binding protein homolog 2 & 3 (PF01237) 12
Glycoproteins metabolism **13**
Glucosyltransferases 13
Glucosyltransferase 32 (PF04488, OCH1/SUR1) 13
Sterol 3-beta glucosyltransferase (PF03033, ATG26) 14
Sialidase (PF13088, NEU2) 15
Complex sphingolipid metabolism **16**
Neutral sphingomyelin phosphodiesterase (PF03372, SMPD2/3) 16
Alkaline ceramidase (PF05875, ASAH3) 17
Phospholipid phosphatase 1 (PF01569, PLPP1) 18
Dated ML trees **19**
Diacylglycerol kinase (PF00781, DGK1) 19
Diacylglycerol O-acyltransferase 1 (PF03062, DGAT1/SOAT1) 20

Fatty acids metabolism

Desaturases

Acyl-CoA 6-desaturase (PF00487, FADS2)

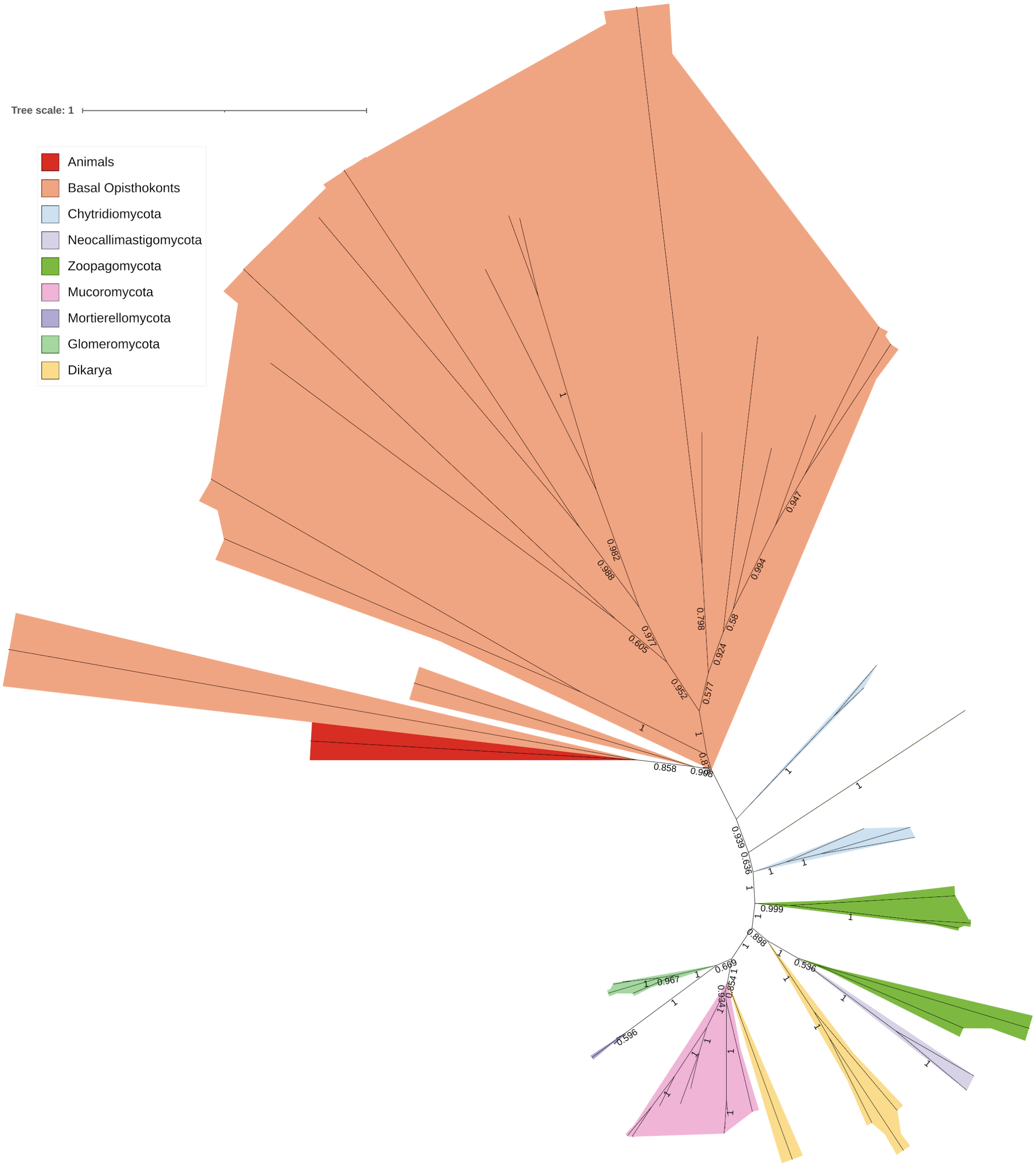

Acyl CoA-desaturase/delta-9 fatty acid desaturase (PF00487, SCD5)

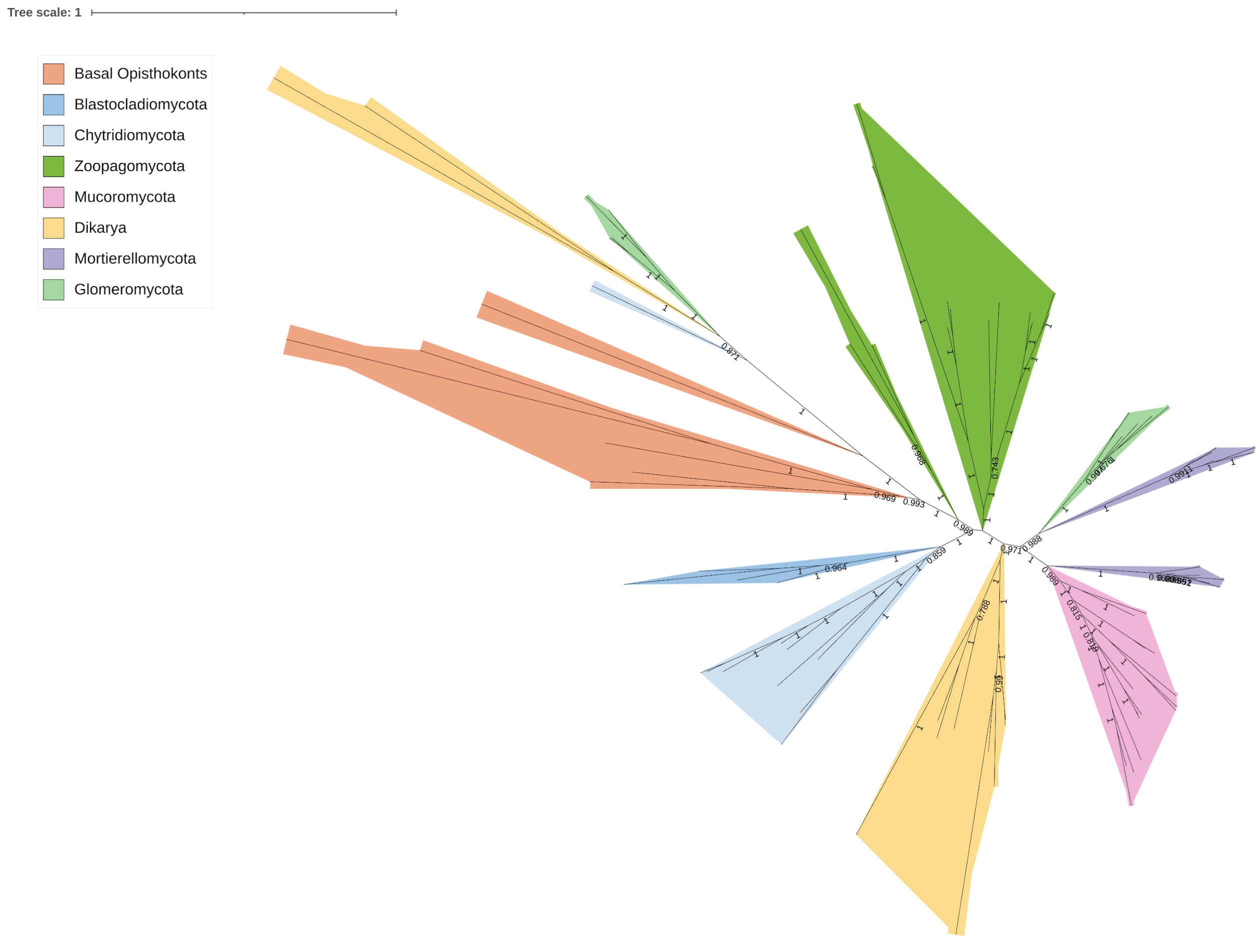

Ceramide biosynthesis

Serine palmitoyltransferase (PF00155, SPT2/3)

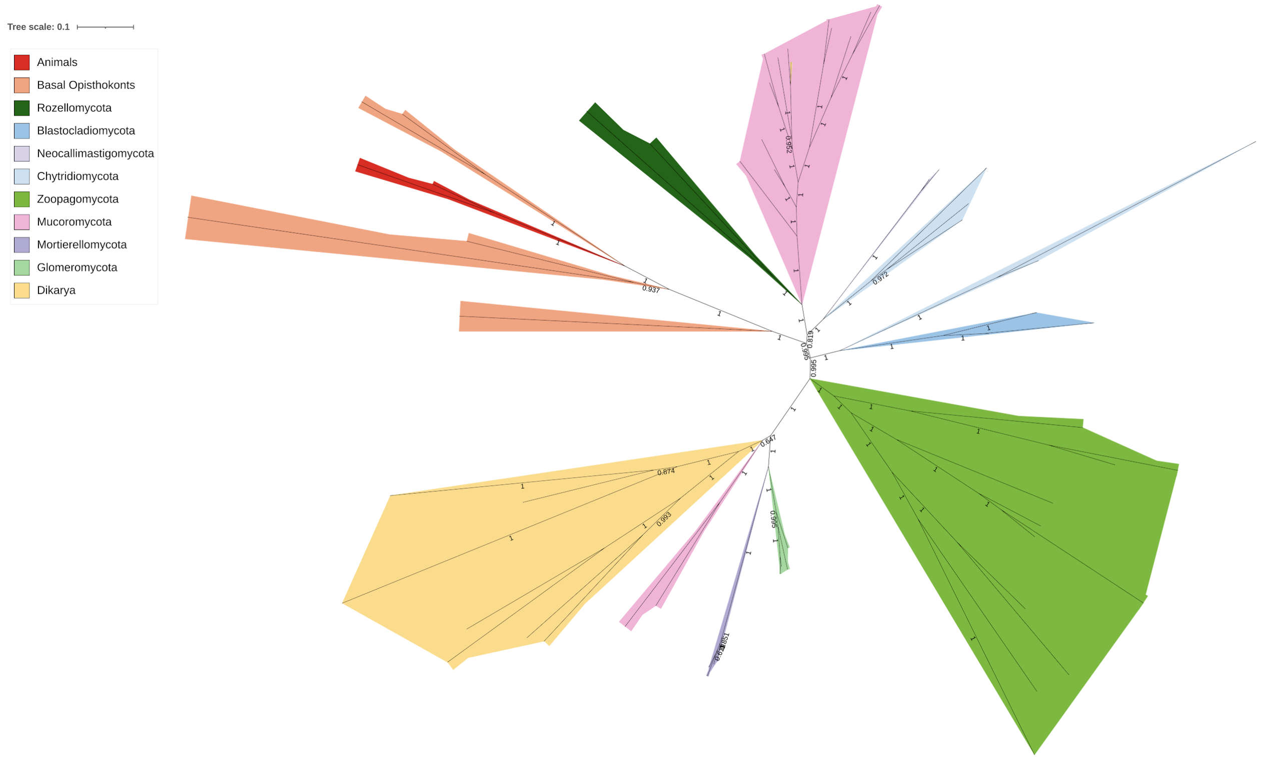

Sphingomyelin synthase (PF14360, SMS)

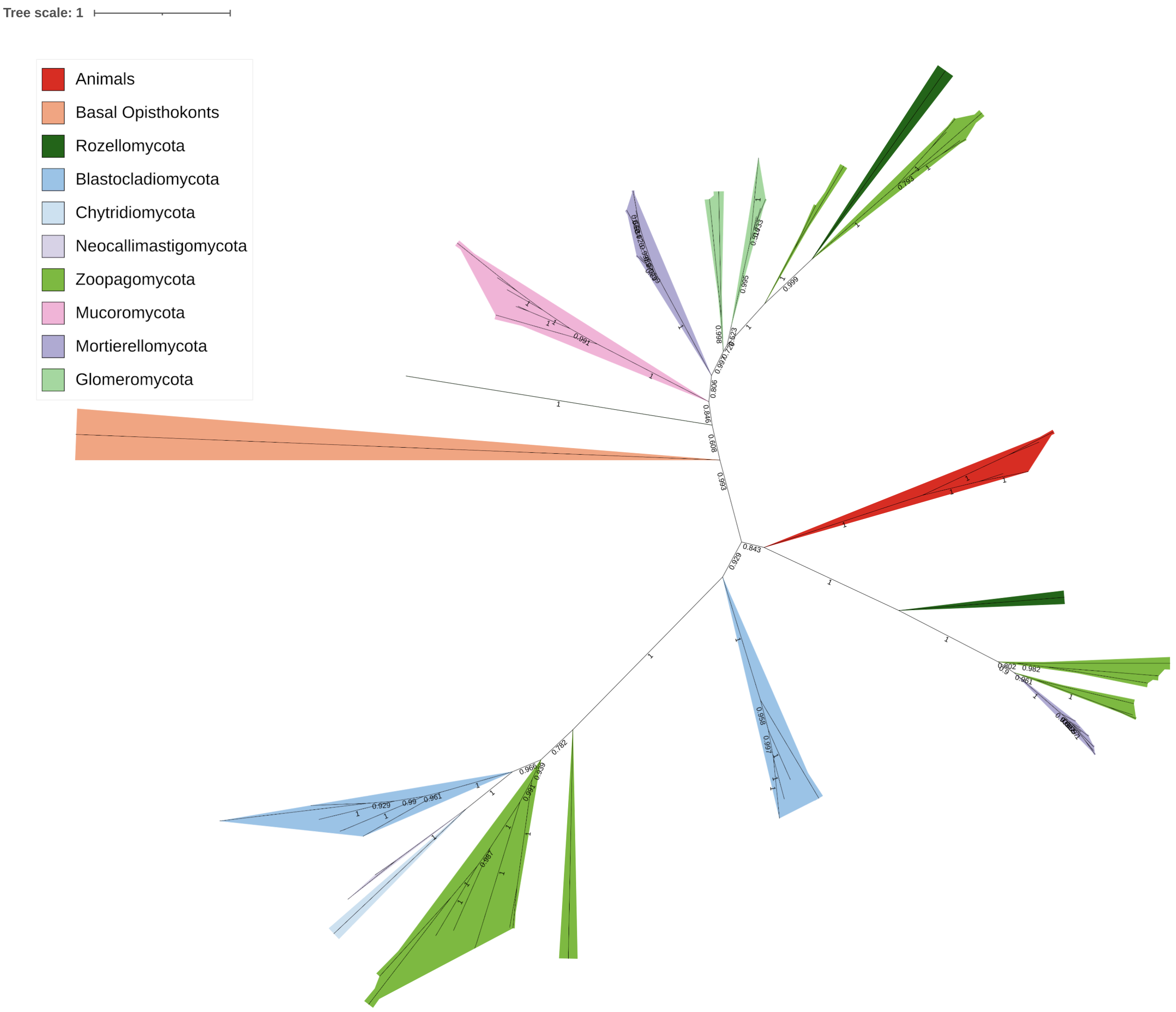

Elongases

Very long chain fatty acid elongases (PF01151, ELOVL)

Very long chain fatty acid elongase (PF01151, ELOVL) forms four subfamilies: very long chain fatty acid elongase 5 (ELOVL5), fatty acid elongase 3 (ELOVL3) and *S. cerevisiae* fatty acid elongase 1, 2 and 3 (ELO1-2/SUR4) as well as unknown fatty acid elongase (ELO)

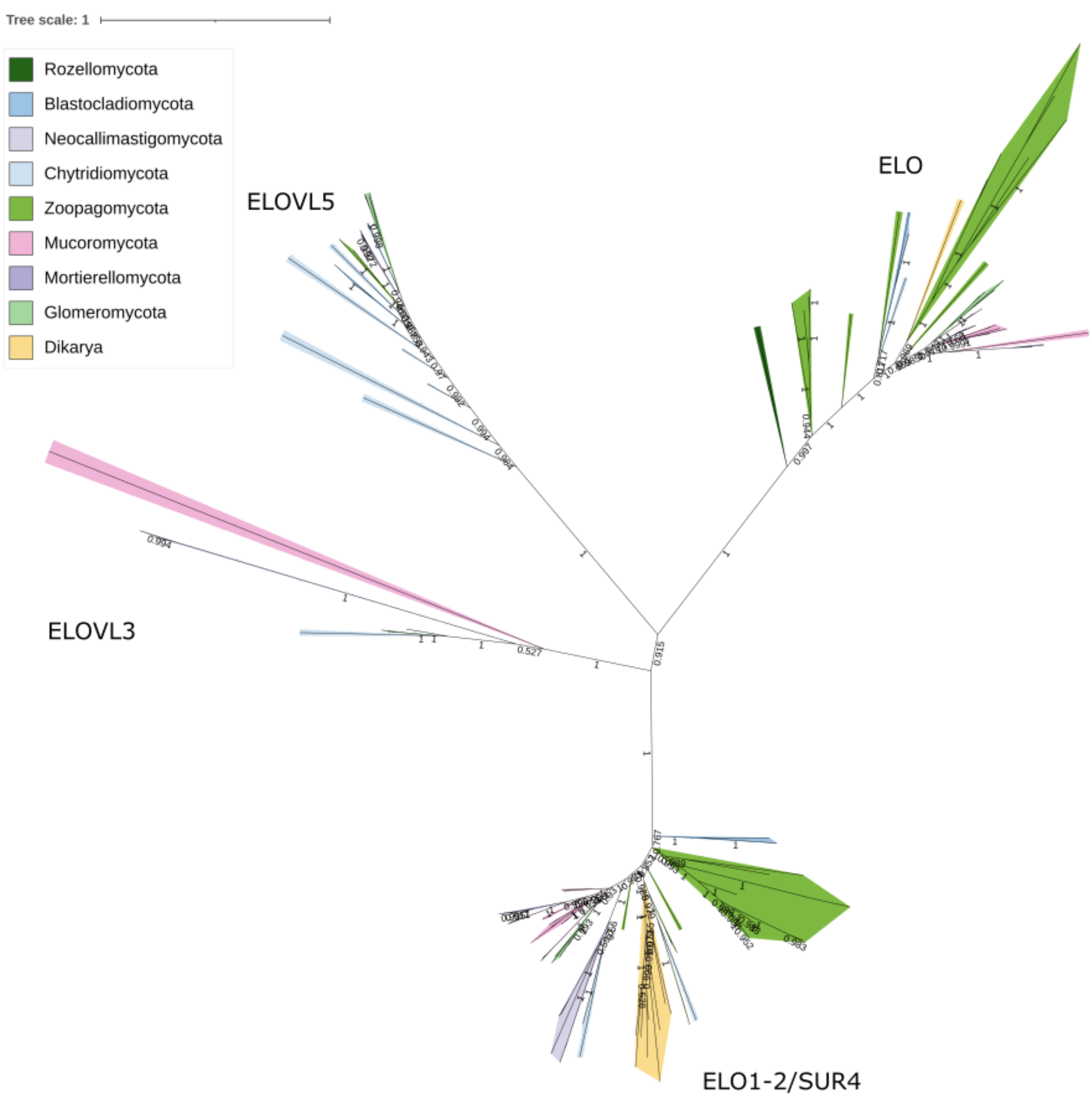

Acyltransferases

Diacylglycerol O-acyltransferase 2 (PF03982, DGAT2)

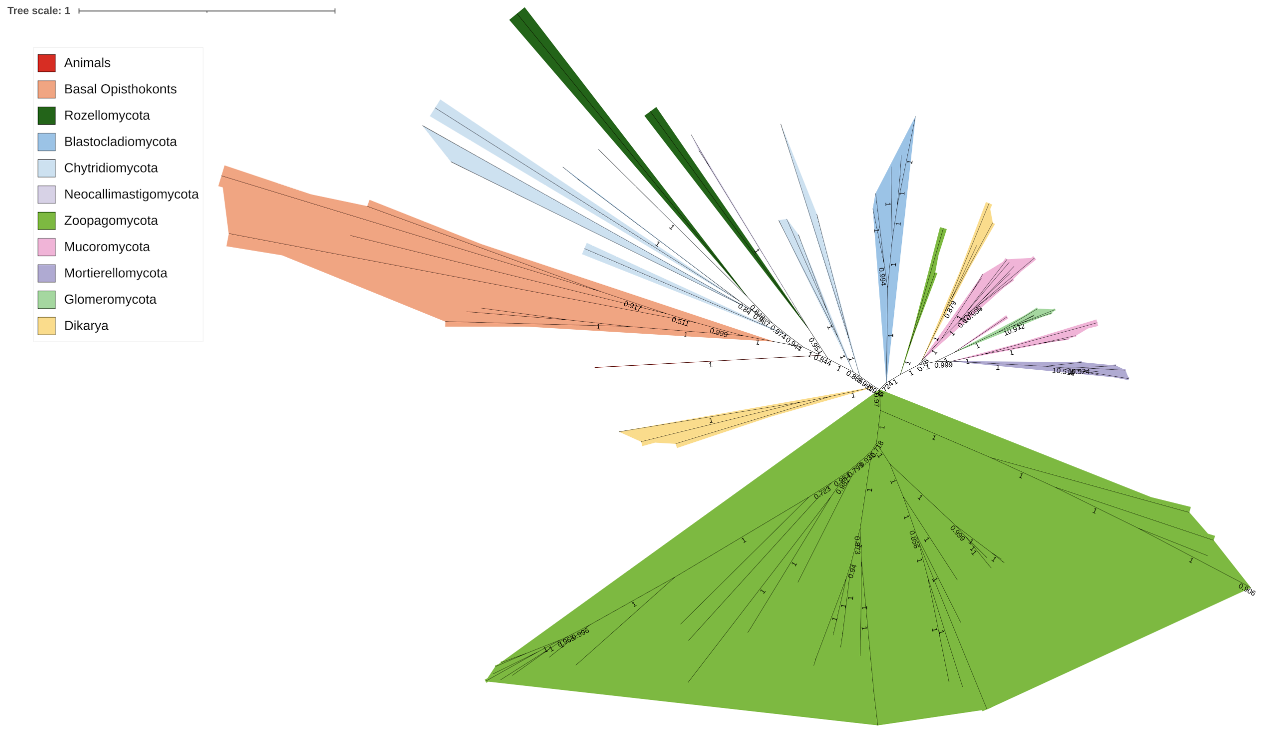

Diacylglycerol O-acyltransferase 1 (PF03062, DGAT1/SOAT1)

Diacylglycerol O-acyltransferase 1 (PF03062, DGAT1/SOAT1) forms two subfamilies: diacylglycerol O-acyltransferase 1 (DGAT1) and sterol O-acyltransferase 1 (SOAT1)

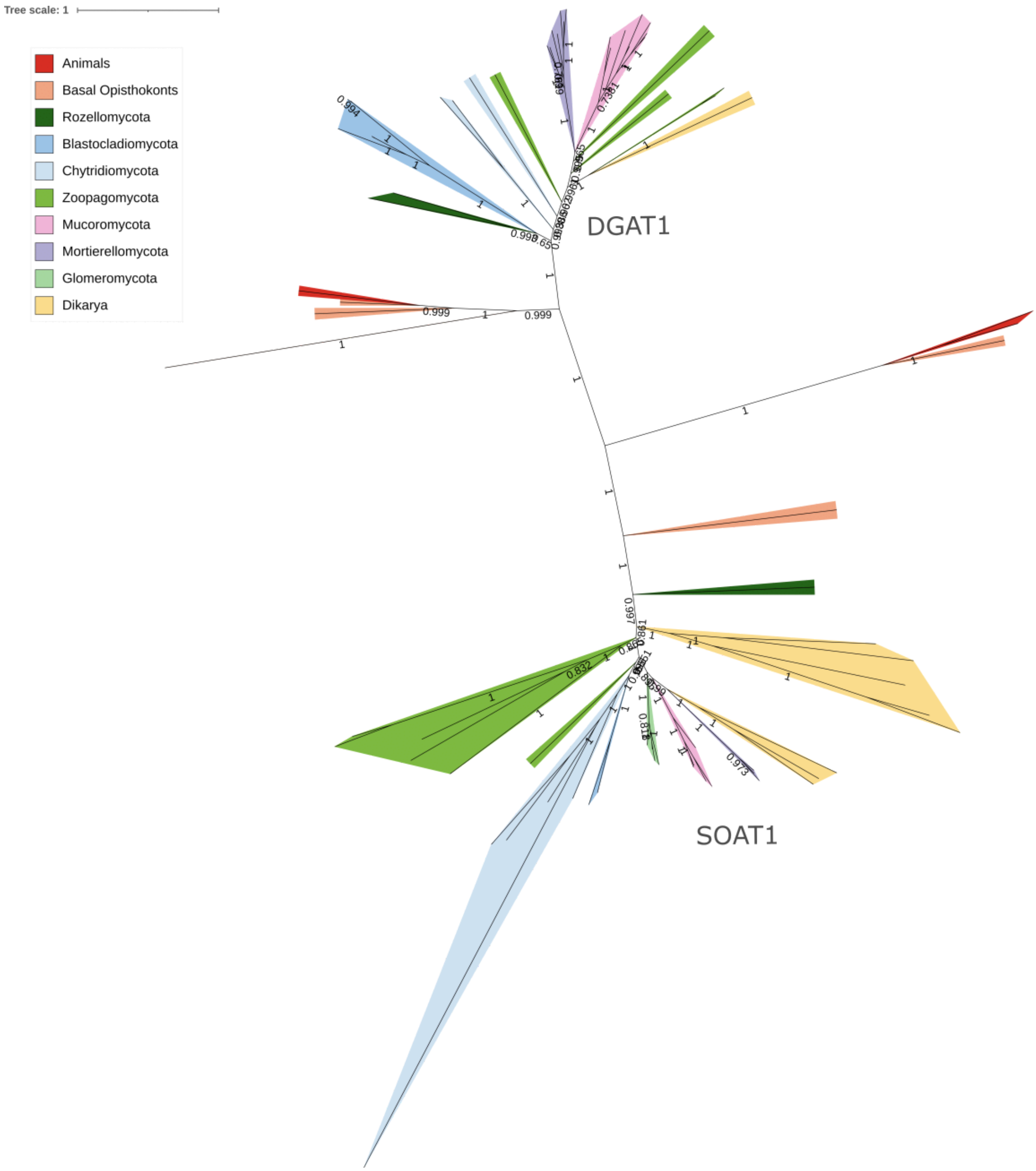

Wax ester synthase (PF03007, WSD1)

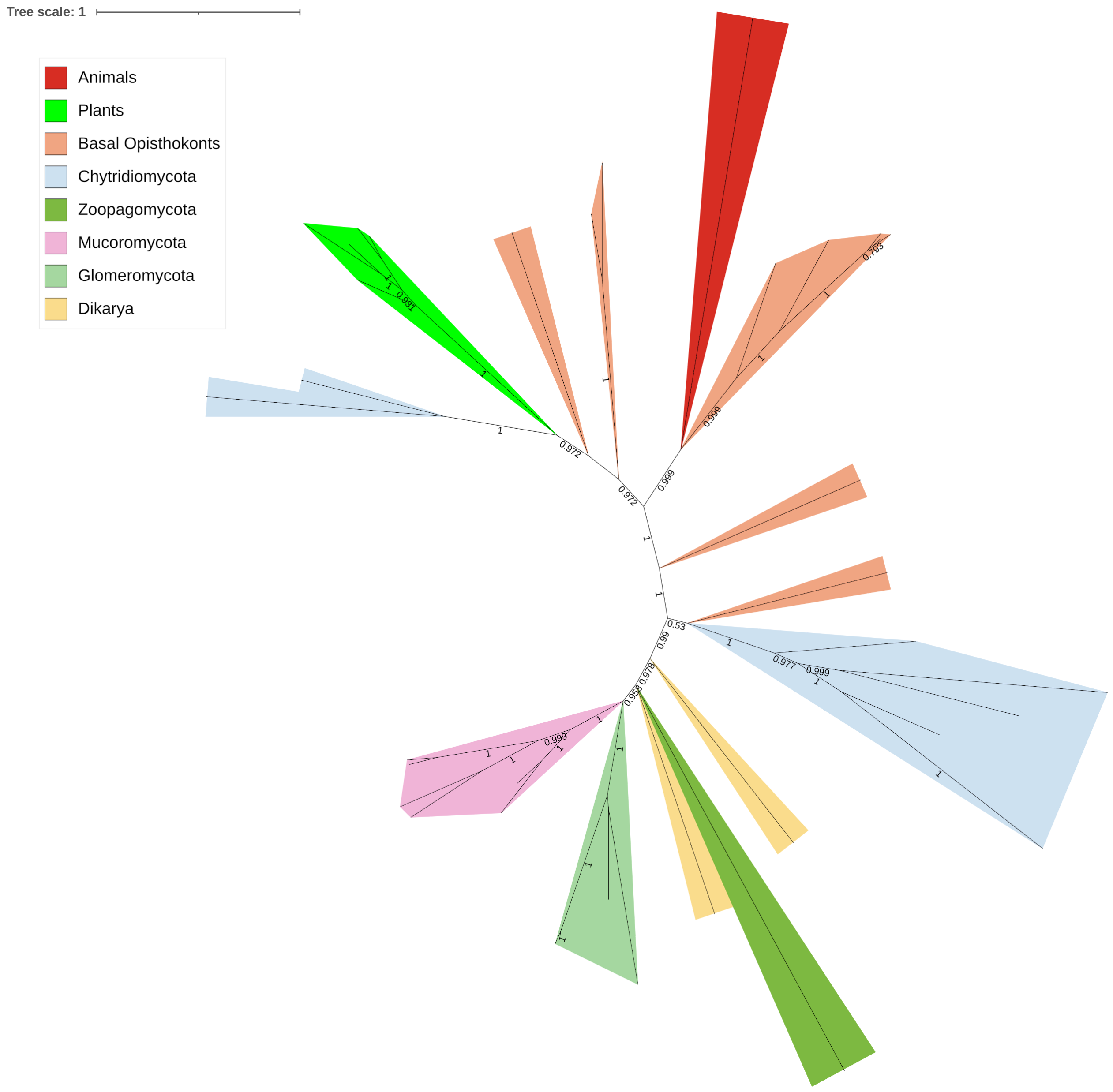

Lipases

Lysophospholipase (PF01735, PLA2G4A)

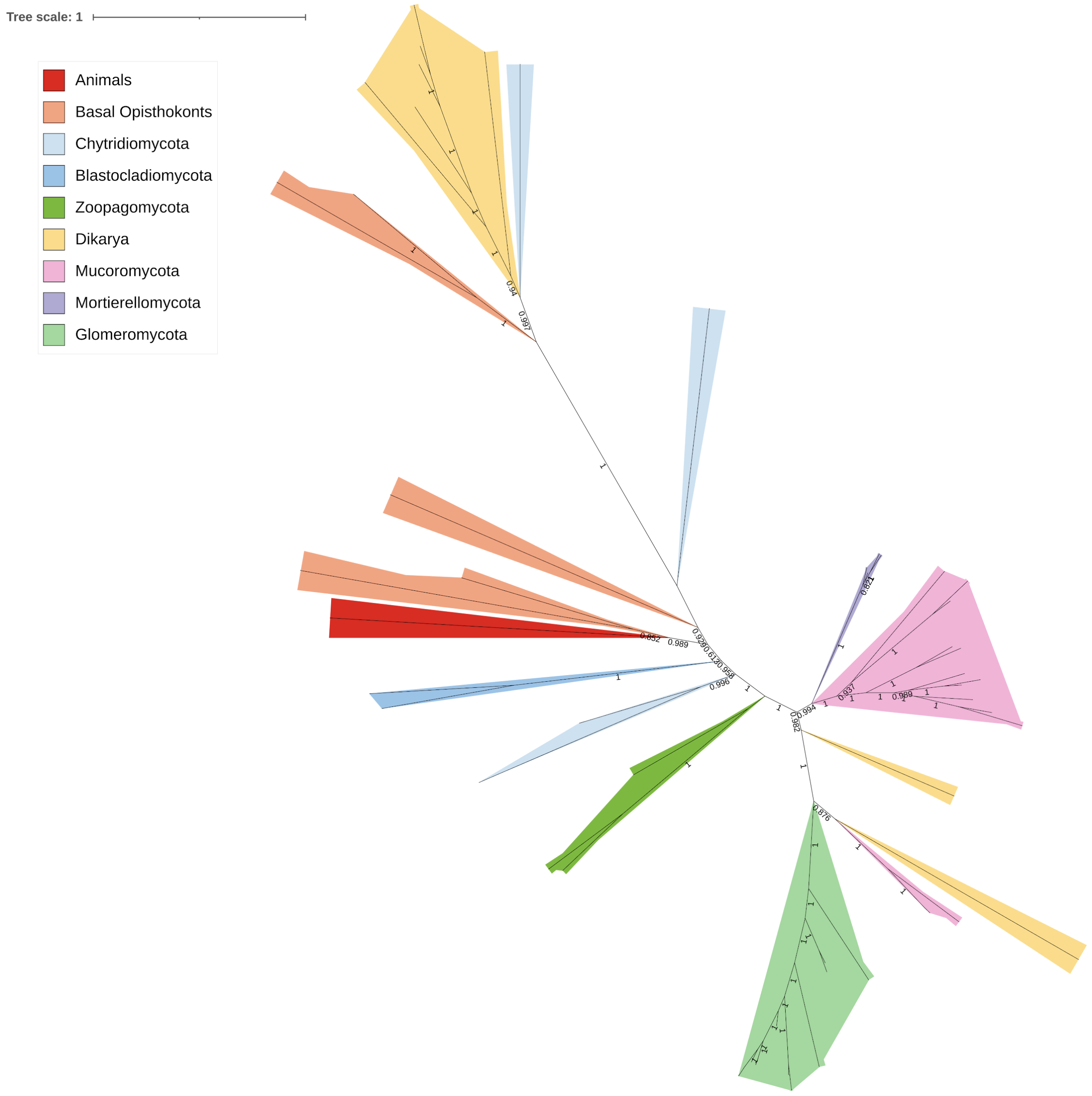

Lipids transfer

Oxysterol-binding protein homolog 4 (PF01237, KES1)

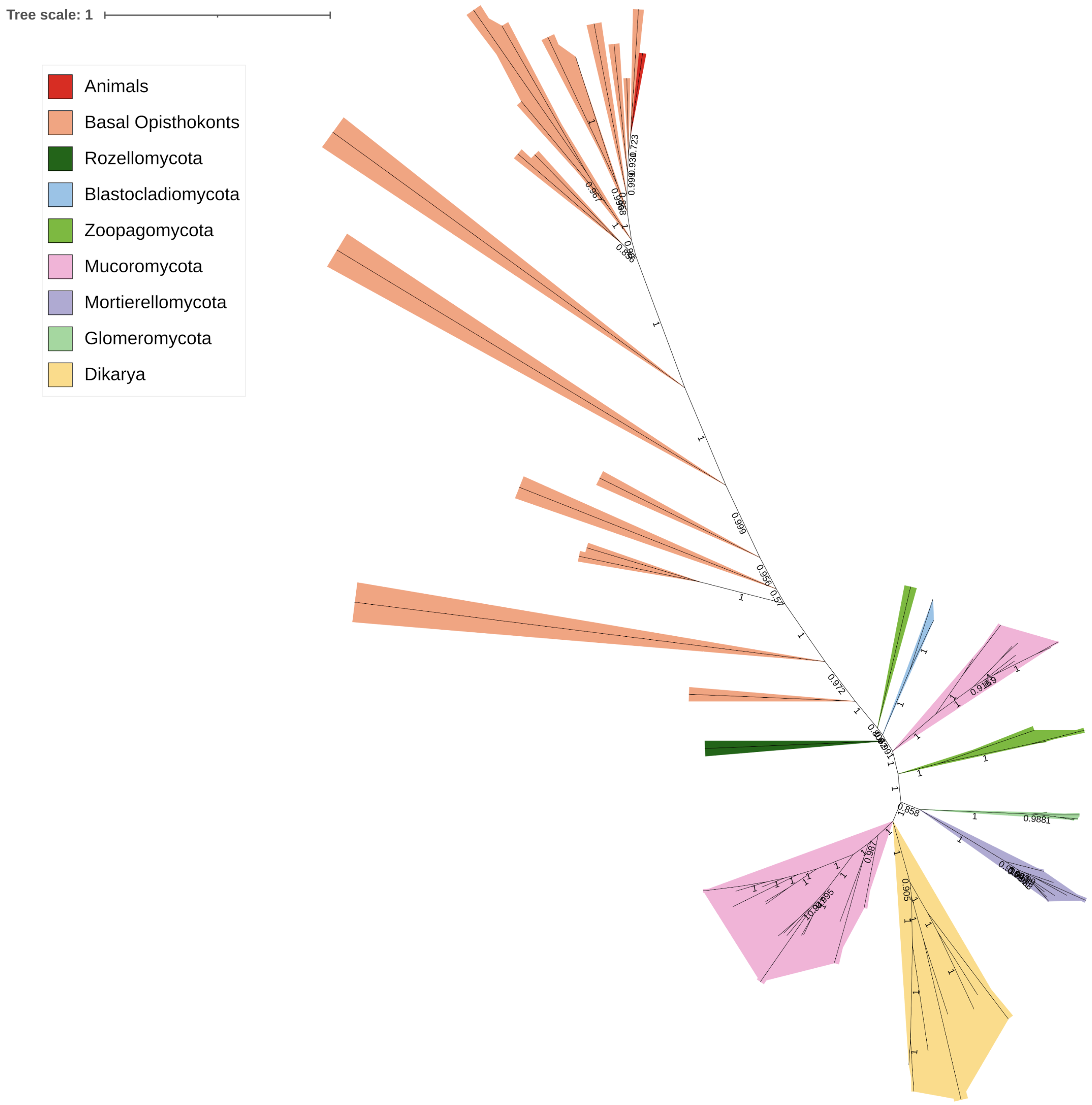

Oxysterol-binding protein homolog 2 & 3 (PF01237)

Oxysterol-binding protein homolog 2 and 3 (PF01237) forms two subfamilies OSH2 and OSH3

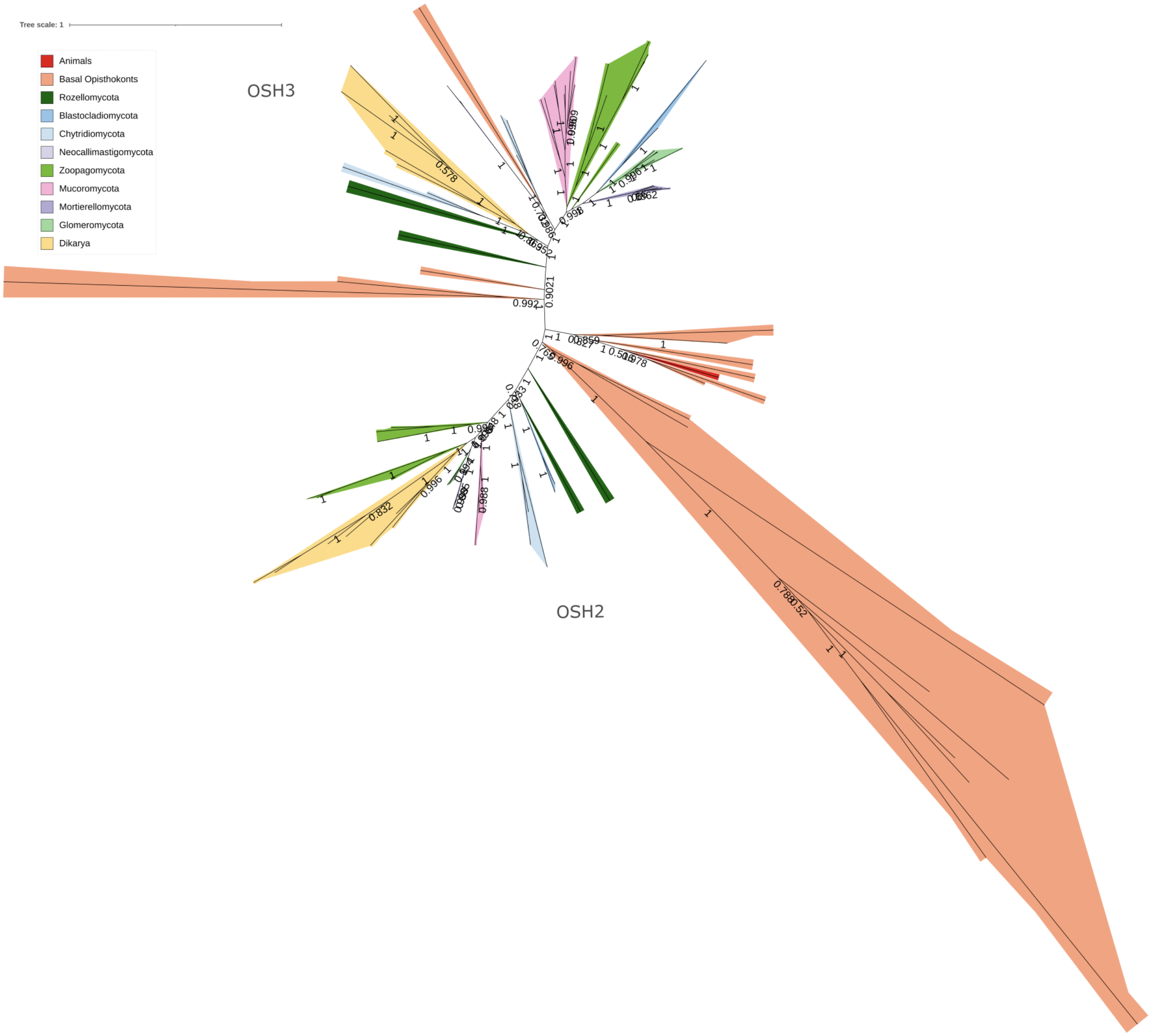

Glycoproteins metabolism

Glucosyltransferases

Glucosyltransferase 32 (PF04488, OCH1/SUR1)

Glucosyltransferase 32 protein family (PF04488) forms four subfamilies: alpha mannosyltransferase (OCH1), mannosyl phosphorylinositol ceramide synthase (SUR1), unannotated glycosyltransferase OCH1-like and beta-1,3-galactosyltransferase 6 (B3GALT6) homologs)

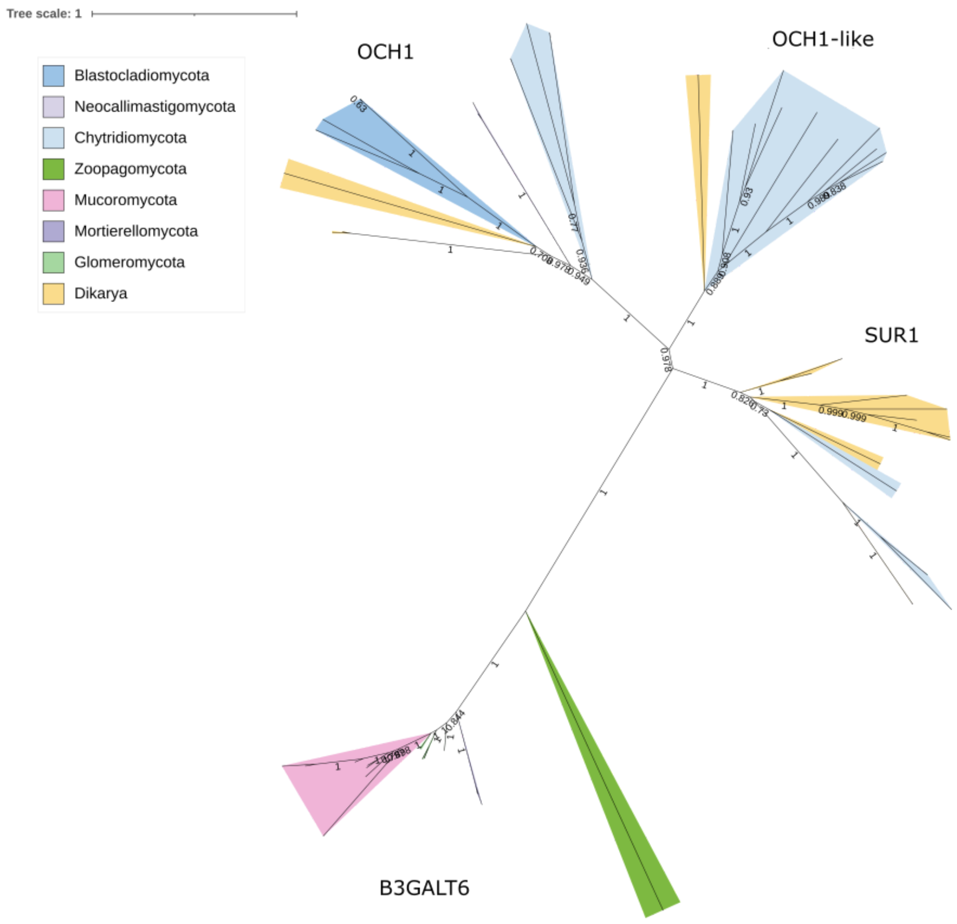

Sterol 3-beta glucosyltransferase (PF03033, ATG26)

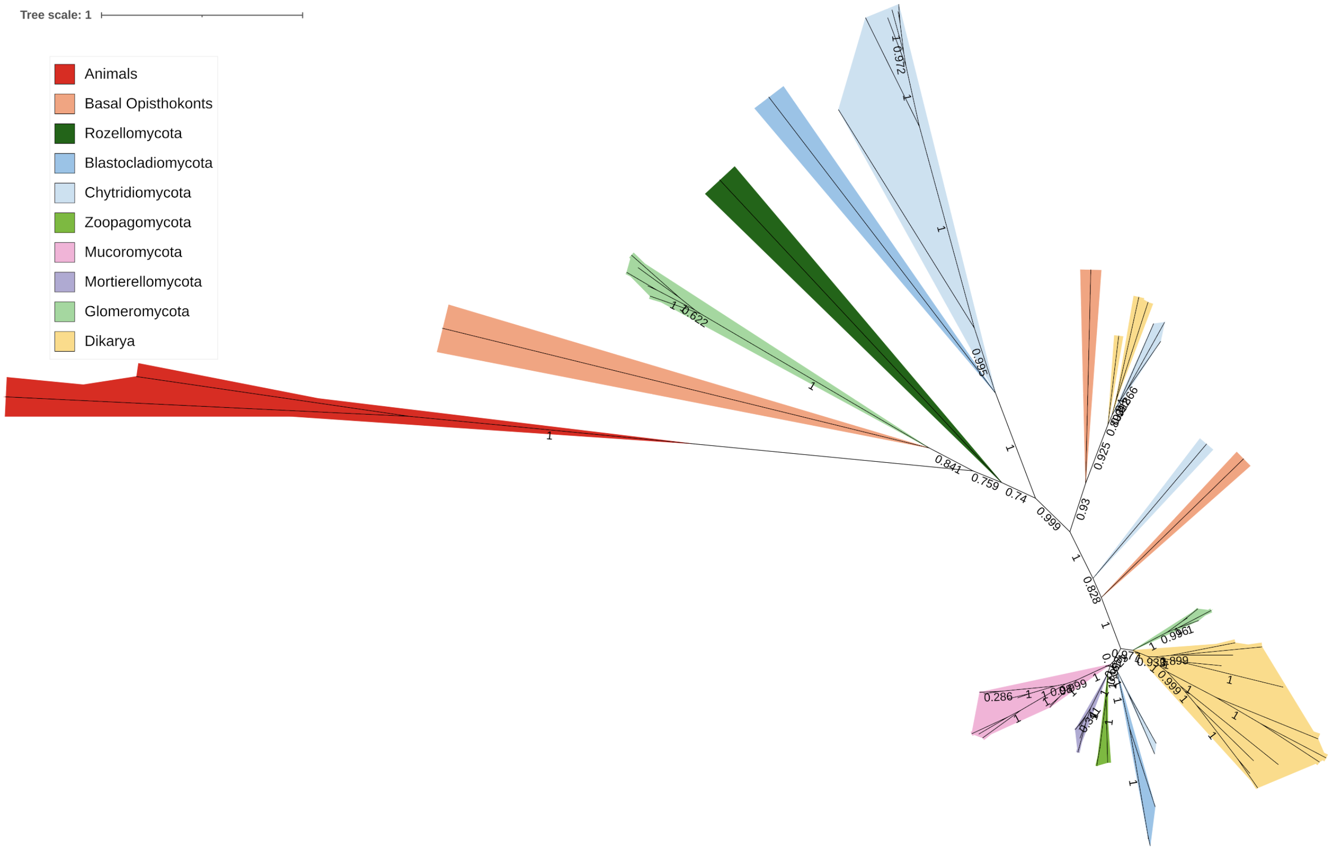

Sialidase (PF13088, NEU2)

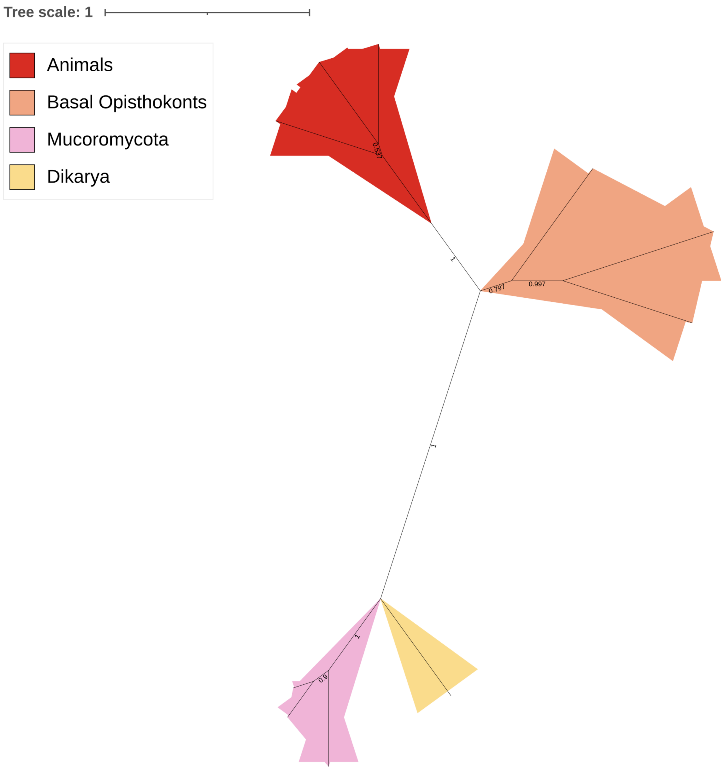

Complex sphingolipid metabolism

Neutral sphingomyelin phosphodiesterase (PF03372, SMPD2/3)

Neutral sphingomyelin phosphodiesterase (PF03372) forms two subfamilies: sphingomyelin phosphodiesterase 2 (SMPD2) and sphingomyelin phosphodiesterase 3 (SMPD3)

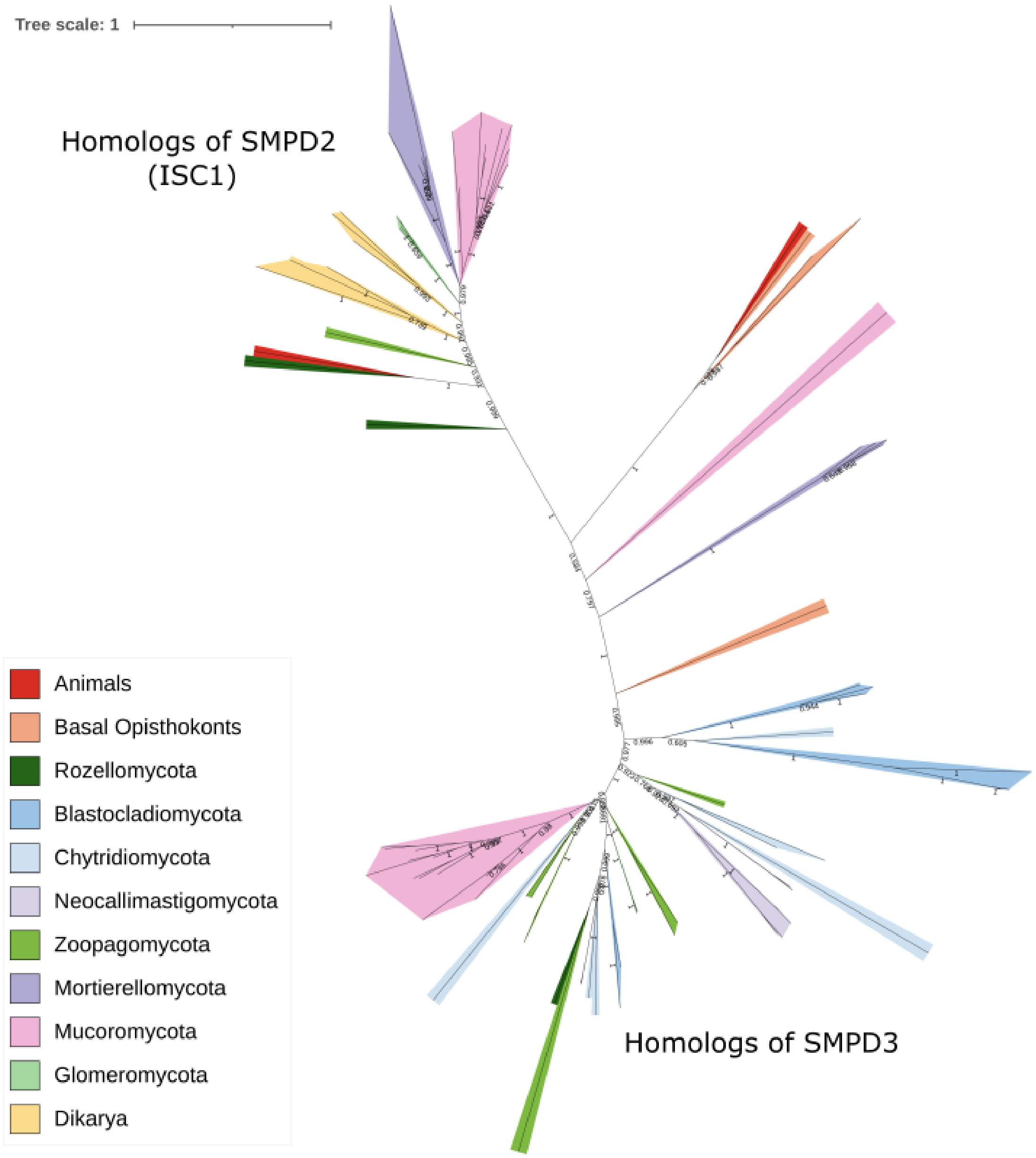

Alkaline ceramidase (PF05875, ASAH3)

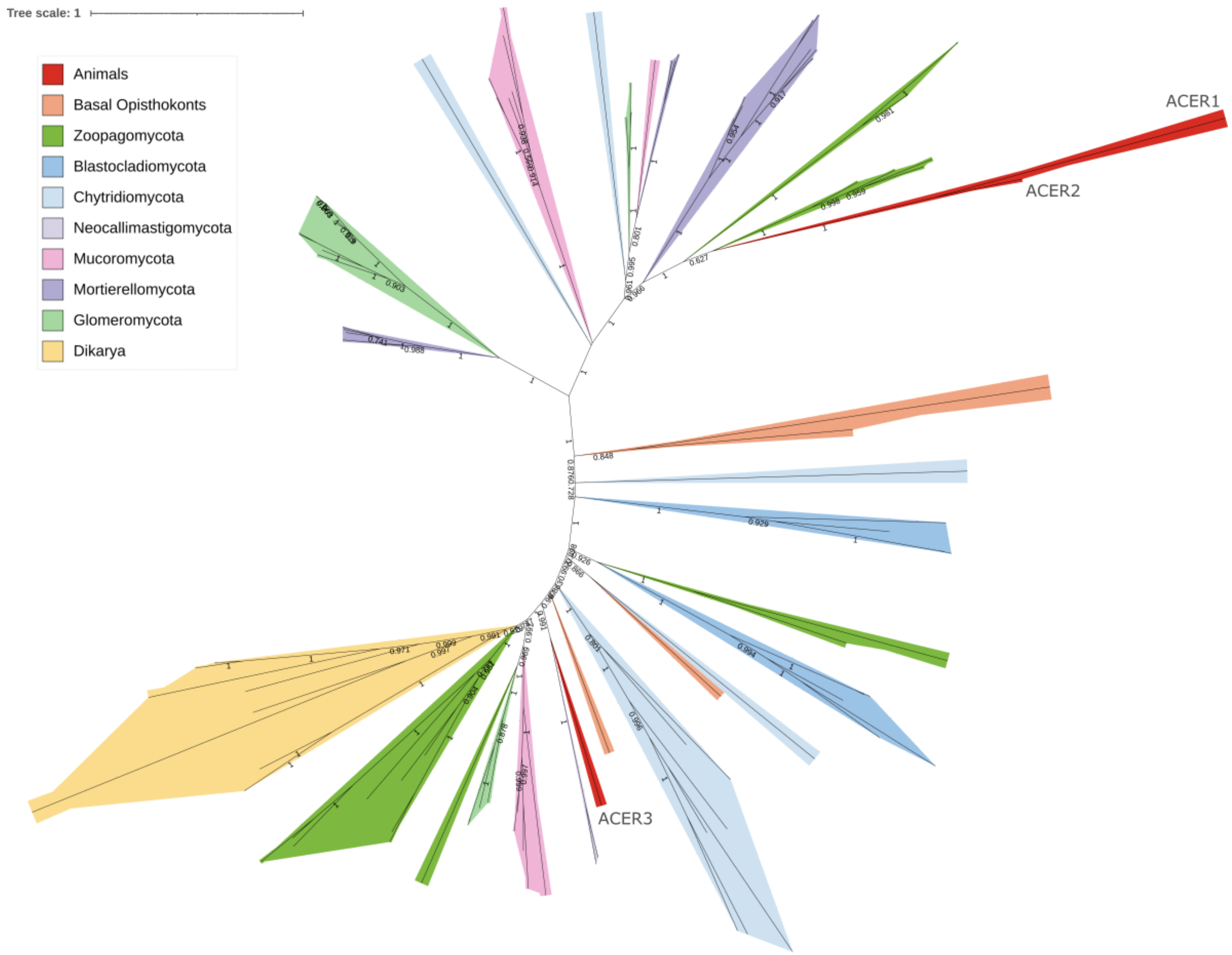

Phospholipid phosphatase 1 (PF01569, PLPP1)

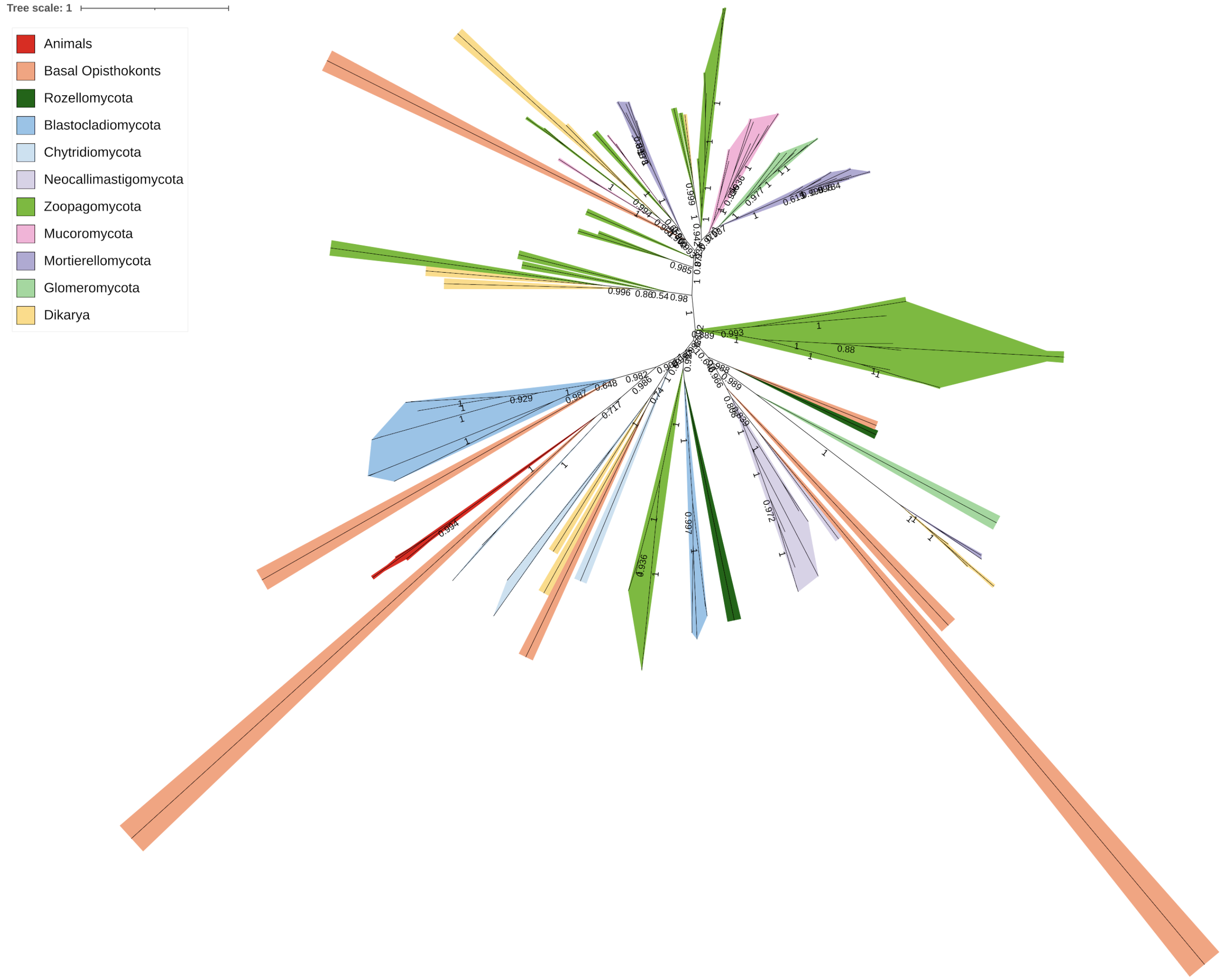

Dated ML trees

Diacylglycerol kinase (PF00781, DGK1)

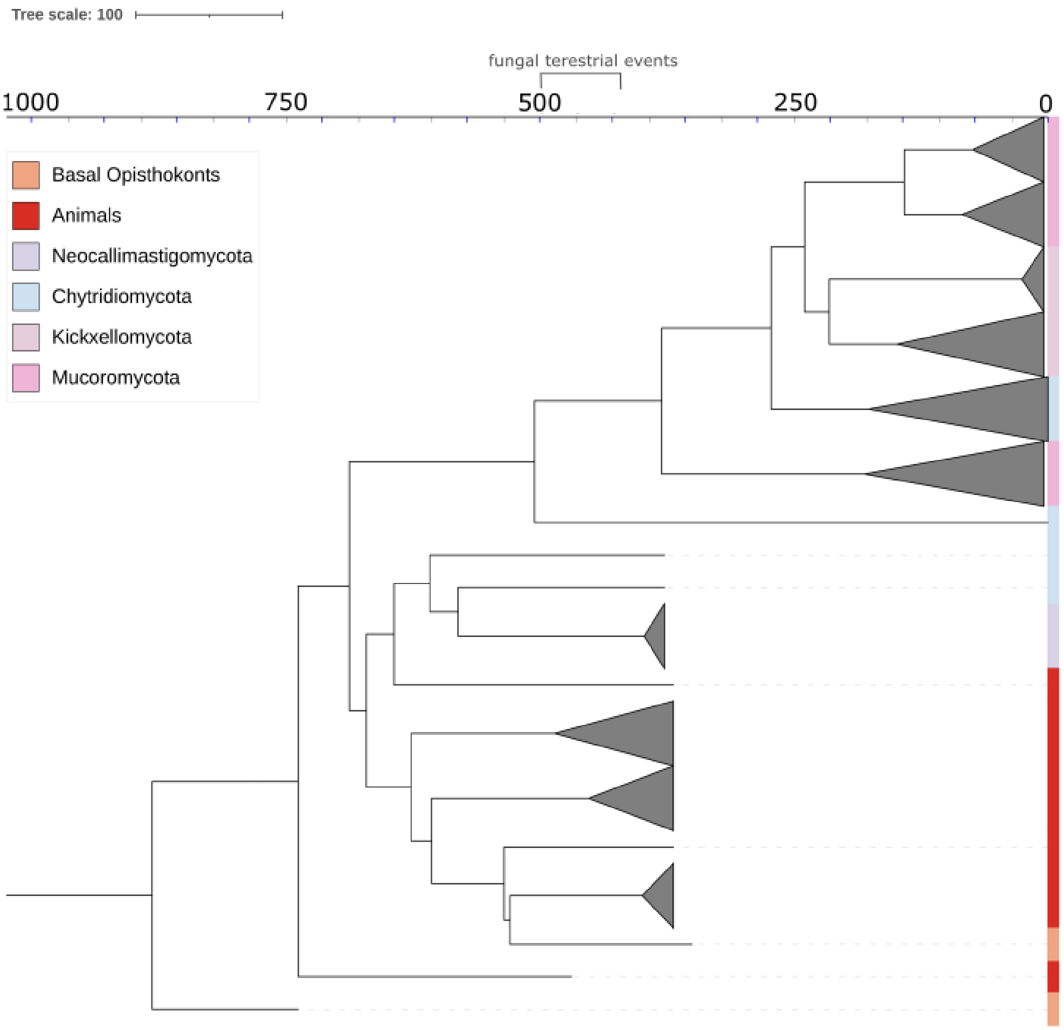

Diacylglycerol O-acyltransferase 1 (PF03062, DGAT1/SOAT1)

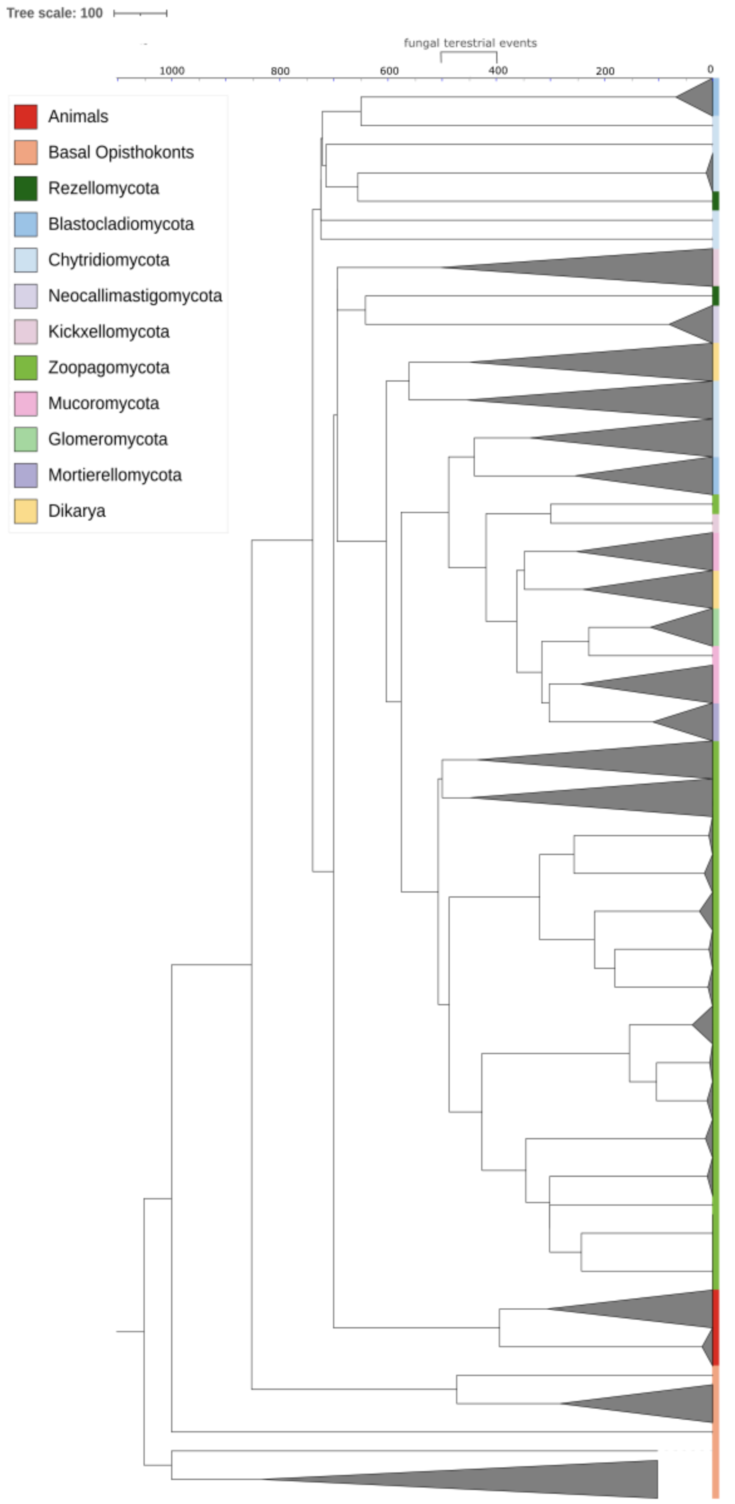

Supplement: Supplementary material 2 — Images of other phylogenetic ML trees and dated trees [file imafungus-17-e177891-s002.pdf]
